# Large-Scale Multi-Omics Studies Provide New Insights into Blood Pressure Regulation

**DOI:** 10.3390/ijms23147557

**Published:** 2022-07-08

**Authors:** Zoha Kamali, Jacob M. Keaton, Shaghayegh Haghjooy Javanmard, International Consortium of Blood Pressure, Million Veteran Program, eQTLGen Consortium, BIOS Consortium, Todd L. Edwards, Harold Snieder, Ahmad Vaez

**Affiliations:** 1 Department of Epidemiology, University of Groningen, University Medical Centre Groningen, 9713 GZ Groningen, The Netherlands; z.kamali@umcg.nl (Z.K.); h.snieder@umcg.nl (H.S.); 2 Department of Bioinformatics, Isfahan University of Medical Sciences, Isfahan P.O. Box 81746-7346, Iran; 3 Division of Epidemiology, Department of Medicine, Vanderbilt University Medical Center, Nashville, TN 37203, USA; jacob.keaton@vumc.org; 4 Center for Precision Health Research, National Human Genome Research Institute, National Institutes of Health, Bethesda, MD 20894, USA; 5 Applied Physiology Research Centre, Cardiovascular Research Institute, Isfahan University of Medical Sciences, Isfahan P.O. Box 81746-7346, Iran; shaghayegh.haghjoo@gmail.com; 6 Regenerative Medicine Research Center, Isfahan University of Medical Sciences, Isfahan P.O. Box 81746-7346, Iran; 7 Division of Epidemiology, Department of Medicine, Vanderbilt Genetics Institute, Vanderbilt University Medical Center, Nashville, TN 37204, USA; todd.l.edwards@vumc.org

**Keywords:** blood pressure, genome, epigenome, gene expression, functional enrichment

## Abstract

Recent genome-wide association studies uncovered part of blood pressure’s heritability. However, there is still a vast gap between genetics and biology that needs to be bridged. Here, we followed up blood pressure genome-wide summary statistics of over 750,000 individuals, leveraging comprehensive epigenomic and transcriptomic data from blood with a follow-up in cardiovascular tissues to prioritise likely causal genes and underlying blood pressure mechanisms. We first prioritised genes based on coding consequences, multilayer molecular associations, blood pressure-associated expression levels, and coregulation evidence. Next, we followed up the prioritised genes in multilayer studies of genomics, epigenomics, and transcriptomics, functional enrichment, and their potential suitability as drug targets. Our analyses yielded 1880 likely causal genes for blood pressure, tens of which are targets of the available licensed drugs. We identified 34 novel genes for blood pressure, supported by more than one source of biological evidence. Twenty-eight (82%) of these new genes were successfully replicated by transcriptome-wide association analyses in a large independent cohort (*n* = ~220,000). We also found a substantial mediating role for epigenetic regulation of the prioritised genes. Our results provide new insights into genetic regulation of blood pressure in terms of likely causal genes and involved biological pathways offering opportunities for future translation into clinical practice.

## 1. Introduction

High blood pressure (BP), also known as hypertension, is a major contributor to morbidity and mortality of heart disease and stroke [1] and, hence, a better understanding of BP regulatory mechanisms is imperative. Individual differences in BP show a robust genetic component of about 50% [2,3], providing opportunities to elucidate the underlying mechanisms of BP regulation through genetic approaches.

Genome-wide association studies (GWAS) have been very successful in discovering genetic determinants of BP [4,5,6,7,8]. The recently published study of over 1 million individuals by Evangelou et al. [9] reported a total of 901 BP-associated loci explaining more than 10% and 27% of the total and single nucleotide polymorphism (SNP)-based heritability of BP, respectively [9].

Successful GWAS analyses merely report SNP–trait associations that do not automatically translate into biological mechanisms. Hence, we need to bridge the functional gap between the identified SNPs and the traits of interest. Otherwise, the relevance of GWAS results can always be criticised. A previously popular follow-up method was to prioritise the genes nearest to GWAS loci. However, the causal genes are not necessarily the nearest genes [10]. Nearby genes harbouring nonsynonymous SNPs may be functionally more relevant than the nearest genes [11]. Other popular follow-up efforts in GWAS studies seek associations with expression levels through expression quantitative trait loci (eQTL) lookups or check overlap of GWAS loci with epigenetic markers [4,6,8,9,12]. However, the causality of these associations is unclear as the observed overlap can be due to the presence of LD between distinct GWAS and regulatory loci [13,14]. Additionally, such lookups simply report SNP–regulatory feature associations, but cannot confirm associations between regulatory features and the outcome trait or disease of interest. Methylome/transcriptome-wide association studies (MWAS/TWAS) yield such regulatory feature–trait associations, but can be driven by nongenetic confounders and do not allow causal inference [13,15,16]. Moreover, while epigenetics has been shown to play a role in BP regulation [17], fine-mapping the downstream genes is challenging, since the target genes may be far from the identified epigenetic markers [13].

The Mendelian randomization (MR) methodology has become popular as it allows investigation of causal relationships, and hence, can be applied to test the causality of methylation/expression–trait associations and link the identified methylation sites to their target genes. However, in the traditional MR approach (i.e., one-sample MR), genetic, methylation/expression, and trait data need to be from the same samples, which is hardly ever available. The recently developed summary data-based MR (SMR) [14] and summary data-based TWAS, i.e., MetaXcan (also called S-Predixcan) [18], approaches only require results files (so called summary statistics), enabling the use of exposure and outcome data from different samples, which substantially increases power and offers opportunities to uncover the hidden links between sequences and consequences.

The previously published large-scale BP GWAS studies [9,19] and post-GWAS functional follow-up studies [20,21] attempted a number of approaches aiming to offer functional annotations of the newly discovered loci. However, there is a need for improving these approaches by following a systematic integrated post-GWAS approach that (i) uses a causal modelling framework rather than relies on simple associations, (ii) uses large-scale datasets rather than data based on small sample sizes, (iii) integrates evidence of as many aspects of biological annotations as possible, and (iv) replicates the functional relevance of the prioritised genes in an independent population.

The recent multi-omics study by Eales et al. [22] investigated effects of BP GWAS loci on the kidney epigenome and transcriptome and yielded 179 unique genes with evidence of causal effects on BP. Despite this success, it means that likely causal genes for the majority of the 901 BP loci remain uncharacterised, implying a need to further elucidate their biological relevance. Post-GWAS investigation of other relevant tissues such as from arteries [7,19] may be key to achieving this.

Another limitation is a lack of large-scale eQTL data from relevant tissues. As cis-eQTL effects are less tissue-specific [23], TWAS discovery analysis can be performed on large-scale blood eQTL data to optimise power, and small eQTL datasets from more relevant tissues can be used to confirm the associations [24].

**Figure 1:**
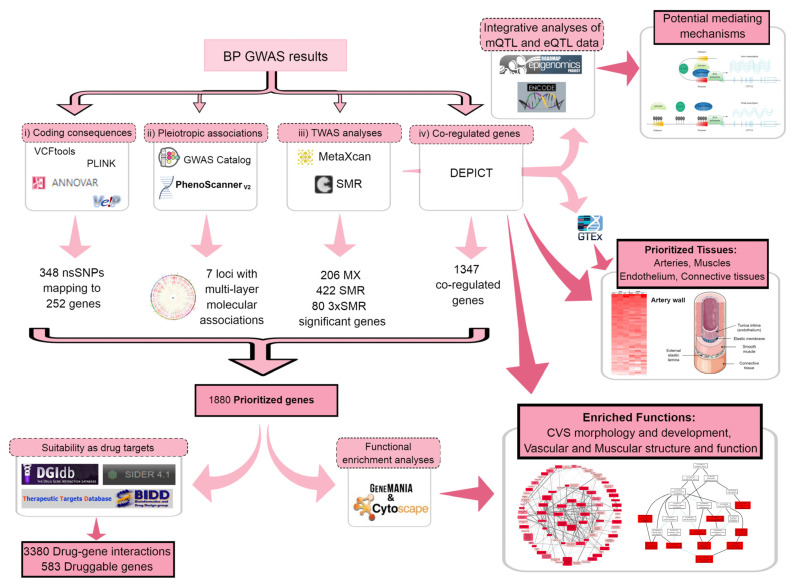
Summary of the methods as well as the main results of prioritization and follow-up analyses using blood pressure GWAS summary statistics. Pink boxes with dashed borders represent methods and their underlying boxes with gray border detail the tools. Pink boxes with solid black borders represent results and their underlying boxes with gray border provide small pictures of results. High resolution figures of heatmap and graphs can be found at the results section. BP: blood pressure; GWAS: genome-wide association study; TWAS: transcriptome-wide association study; nsSNPs: nonsynonymous single nucleotide polymorphisms; MX: MetaXcan; SMR: summary data-based Mendelian randomization; mQTL: methylation quantitative trait loci; eQTL: expression quantitative trait loci; CVS: cardiovascular system.

Here, we performed integrative studies based on our previously published post-GWAS approaches [11,25] with extensions to other molecular layers and also drug–gene interactions. We used comprehensive genomic and transcriptomic data to prioritise likely causal genes and uncover the important mechanisms in genetic regulation of BP independent of and unbiased by previous (nongenetic) knowledge on BP. We first prioritised the likely causal genes by seeking evidence from (i) coding consequences [11,26,27], (ii) multilayer associations with molecular traits [28], (iii) TWAS analyses [14,18], and (iv) gene coregulation surveys [29]. Next, we performed tissue scans using different approaches [14,18,29,30] to find the most relevant tissues for BP. Then, we performed confirmatory TWAS in the most relevant tissues for highly prioritised genes. Finally, we checked the prioritised genes against drug–gene interaction databases to highlight potential clinical relevance [31,32,33] and investigated mediating mechanisms [13,29,34,35,36,37] connecting DNA to BP, using epigenetic data as well as functional predictions (Figure 1).

## 2. Results

### 2.1. Gene Prioritization

#### 2.1.1. Coding Consequences of BP Loci

In silico sequencing returned 63,049 SNPs that are in LD (r^2^ > 0.50) with the 901 GWAS SNPs (gSNPs) (Supplementary Table S1); 348 SNPs, including 43 gSNPs and 305 linked SNPs, were annotated as nonsynonymous (ns)SNPs, altogether mapping to 252 genes, 13 of which had not been previously reported for BP. Thirty-six out of the 348 nsSNPs, including six gSNPs, were predicted to have deleterious and possibly/probably damaging effects by the Sorting Intolerant from Tolerant (SIFT) algorithm and the Polymorphism Phenotyping (Polyphen) scores, respectively (Supplementary Table S2).

#### 2.1.2. Seeking Pleiotropic Associations of BP Loci with Different Traits

In silico lookups of pleiotropic associations of BP loci and other GWAS catalog traits returned 2088 SNPs mostly associated with cardiovascular-, lipid-, anthropometric-, and psychiatric-related traits. Furthermore, we observed a pleiotropic effect on BP and aortic root size as well as aortic stiffness (Supplementary Table S1).

**Figure 2:**
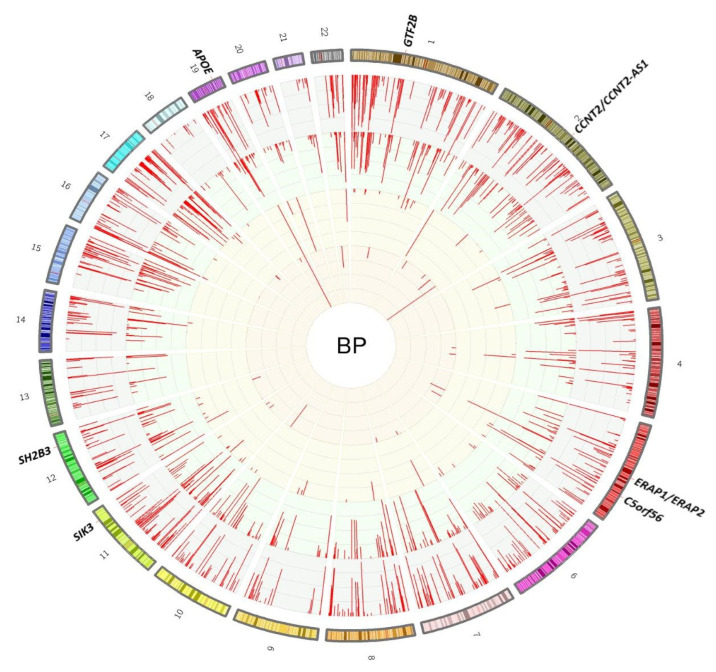
In silico lookup of BP loci associated with different molecular traits at the genome-wide significance level (*p* < 5 × 10^−8^; from QTL tests). The outer layer represents the genomic position of variants with QTL associations and is split up into 22 chromosomes with banded colours showing the cytobands. The inner layers with red lines (from the outer one to the inner one) are as follows: DNA methylation, transcription, protein, and metabolite levels. The height of the red lines is representative of −log_10_(P). The seven assigned loci are those with evidence of simultaneous associations with all four molecular traits.

Phenoscanner lookups of BP GWAS loci showed 749, 569, 64, and 23 associations with DNA methylation (mQTL), gene expression (eQTL), protein levels (pQTL), and metabolite levels, respectively. Strikingly, variants at seven genes were simultaneously associated with all four molecular traits (Figure 2). These included *GTF2B*, *CCNT2/CCNT2-AS1*, *ERAP1*/*ERAP2*, *C5orf56*, and *SIK3*. The two other genes, *SH2B3* and *APOE*, also have nonsynonymous SNPs linked with BP loci (r^2^ > 0.5) and have been highlighted as hotspots for a large number of gene expressions and metabolite levels, respectively (Supplementary Table S3).

#### 2.1.3. Transcriptome-Wide Association Study (TWAS) Using Summary Statistics

TWAS analyses by MetaXcan (MX) based on the Depression Genes and Networks (DGN) whole blood transcriptome data returned 544, 643, and 450 significant genes for systolic, diastolic, and pulse pressure, respectively. By excluding genes with low prediction performance, 396, 475, and 338 significant genes remained for the three blood pressure traits, respectively (Supplementary Table S4a–c). After removing duplicates across traits, a total of 761 BP-significant genes remained, of which 206 showed acceptable evidence of colocalised signals (see Methods). Twenty-one of these identified genes had not been previously reported for BP.

SMR analyses using eQTL data from the eQTLGen consortium yielded 574, 639, and 485 genes, the expression levels of which were significantly associated with systolic, diastolic, and pulse pressure (SBP, DBP, and PP), respectively. By selecting genes with no evidence of confounding by linkage based on the heterogeneity test (P_HEIDI ≥ 0.01), 205, 208, and 176 significant genes remained for SBP, DBP, and PP, respectively (Supplementary Table S5a–c). After removing duplicates, we ended up with a list of 422 likely causal genes for BP traits in total. Out of the genes identified by SMR, 91 had not been previously reported for BP.

#### 2.1.4. Determining Coregulated Genes within BP-Associated Loci

Data-driven Expression Prioritised Integration for Complex Traits (DEPICT) identified 721, 817, and 712 genes within the constructed loci for SBP, DBP, and PP, respectively, which were coregulated more than expected by chance (FDR ≤ 0.01). Merging the three gene lists resulted in a total of 1347 prioritised genes for BP traits (Supplementary Table S6a–c). A total of 210 of the significantly coregulated genes had not been previously reported for BP.

### 2.2. Tissue Prioritization

**Figure 3:**
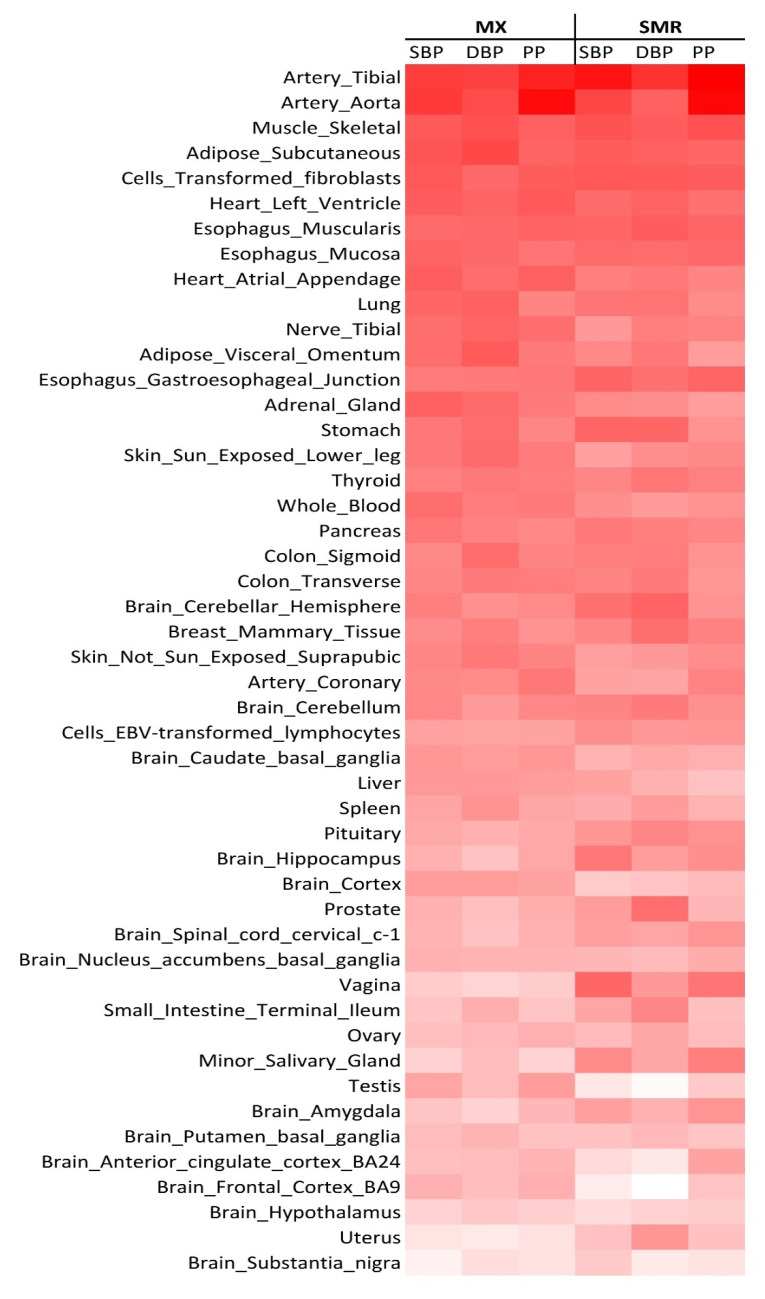
Average Z squares of the genes’ associations with BP traits across 48 GTEx tissues using both MetaXcan (MX) and summary data-based MR (SMR) TWAS. Colour intensity set to higher values. The order of tissues is based on the mean of the average Z squares of the genes across BP traits and the two TWAS approaches.

Our tissue prioritization based on TWAS (i.e., MX and SMR) across 48 tissues from the Genotype-Tissue Expression (GTEx) database returned tibial artery and aorta as well as muscles as the top prioritised tissues (Figure 3). It is noteworthy that the adrenal gland, a well-known focus point in hypertension mechanism, was ranked 14th (top 30%) among the prioritised tissues. In line with the TWAS results, DEPICT showed the most significant levels for arteries and muscle tissues (Supplementary Table S7). 

### 2.3. Functional Follow-Up of the Genetically Prioritised Genes

Merging the prioritised gene sets, i.e., including (i) 252 genes with coding consequences (Supplementary Table S2), (ii) seven genes from multilayer associations with molecular traits (Supplementary Table S3), (iii) 522 genes with expression levels associated with BP (Supplementary Tables S4a–c and S5a–c), and (iv) 1347 genes coregulated with other genes from BP-associated loci (Supplementary Table S6a–c), resulted in a final list of 1880 prioritised genes for all the BP traits combined (Supplementary Table S8). This list, as well as the trait-specific subset lists including 1041 genes for SBP, 1144 genes for DBP, and 957 genes for PP, were then used for downstream functional analyses (Figure 1).

#### 2.3.1. Suitability of the Prioritised Genes as Drug Targets

Querying the drug–gene interaction database (DGIdb) with the list of 1880 prioritised genes returned 364 drugged genes (Supplementary Table S9a–c) as well as 583 druggable genes (Supplementary Table S9d). The 364 drugged genes interact with 2122 drugs.

Our query of the drugs with the primary indication of hypertension or hypotension in the therapeutic target database (TTD) returned 100 drugs, of which 79 interacted with 42 prioritised genes (Supplementary Table S9a). Fisher’s exact test showed enrichment of hyper-/hypotension drug targets among our list of prioritised genes (*p*-value < 2.2 × 10^−16^; fold change of ~9.7). When restricting to genes with evidence for more than one BP trait, the fold change even increased to 12.3.

Besides the 42 prioritised genes targeted by BP therapeutics, 322 of our prioritised genes were targets of non-BP therapeutics and hence could be candidates for drug repurposing. Furthermore, targeting drugs of the 159 prioritised genes showed hyper/hypotensive adverse effects (Supplementary Table S9b). The information for other interacting drugs is provided in Supplementary Table S9c.

#### 2.3.2. Functional Enrichment Analyses of the Prioritised Genes

**Figure 4:**
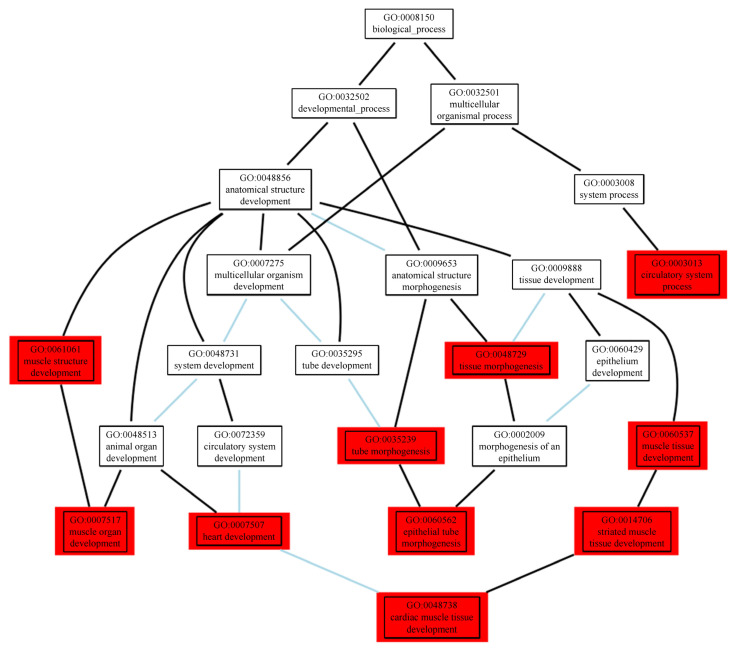
Hierarchical relationships of the top 10 enriched pathways for BP in a Gene Ontology (GO) tree. Black connectors mean “is a” and blue connectors mean “part of”. Coloured in red are the top 10 significantly enriched (FDR < 0.01) GO terms.

Our functional and network analysis based on the final list of 1880 prioritised genes (see methods) using Cytoscape extended by the GeneMania plugin resulted in 313 significant biological pathways (q-value < 0.01). Since the substantial overlap between the Gene Ontology (GO) terms makes the results correlated, we also investigated significant terms in the GO tree to find super terms under which significant results converge (Figure 4). We also categorised and colour-coded the full results table (Supplementary Table S10a). More than one-third of all the significant terms and, in particular, the top 5% of the list, point towards development, morphogenesis, and anatomical structure of the cardiovascular system, e.g., cardiac muscle, muscle structure, muscle cells, striated muscle, muscle system, muscle organ, muscle tissue, muscle fiber, smooth muscle cells (SMCs), myoblasts, myofibrils, sarcomeres, actin cytoskeleton, cardiocytes, cardiac cells, cardiac chambers, cardiac ventricles, epithelial tube formation, blood vessels, angiogenesis, etc. The second, less frequent, less significant category of biological pathways point towards development and morphogenesis of the urogenital system, e.g., glomeruli, nephrons, renal tubules, kidney epithelium, ureteric buds, etc. (Supplementary Table S10a). When limiting the input list of prioritised genes to those with at least two sources of evidence (297 genes), the involvement of the arterial wall highlighted by the extracellular matrix (ECM) becomes even stronger (Supplementary Table S11).

After removing the shared significant terms between BP traits, trait-specific analysis revealed ‘developmental growth’ and ‘regulation of anatomical structure size’ for SBP, ‘kidney development’ and ‘metanephric nephron development’ for DBP, and ‘extracellular matrix organization’ and ‘extracellular structure organization’ for PP as the two top findings for each trait (Supplementary Table S10b–d).

Our negative control analyses using five random gene sets returned no significant functions, confirming the robustness of our gene prioritization criteria.

**Figure 5:**
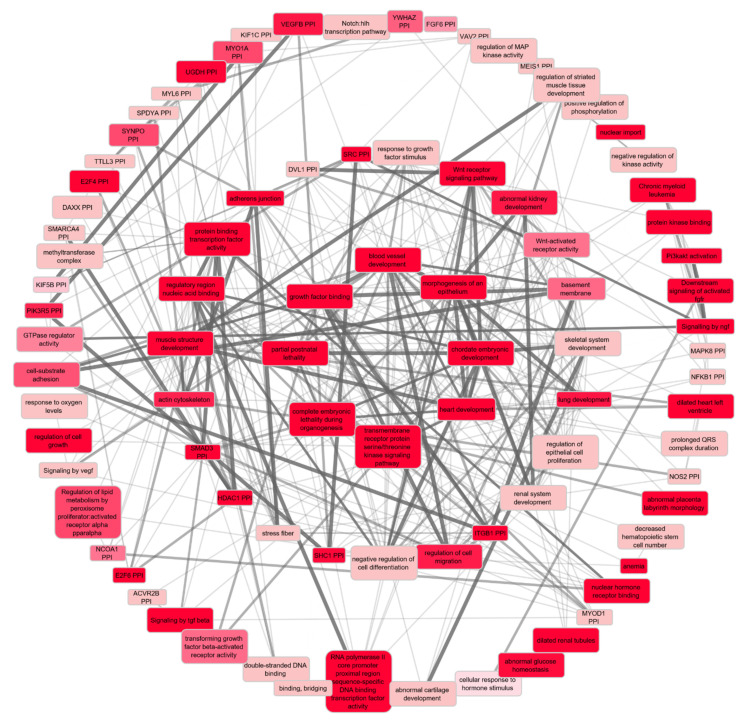
Network plot showing significantly enriched pathways resulting from DEPICT gene set enrichment analysis (FDR < 0.01) and clustered based on coregulation data provided by DEPICT. The outer layer contains pathways with the centrality degree ≤ 10, the middle—with the centrality degree of 10 < x ≤ 20, and the central circle contains pathways with the highest degree (>20), meaning the largest number of connections, which imply their importance in network survival. Among all, heart development has the maximum degree and the lowest *p*-value. Nodes are colour-coded based on the *p*-value.

Gene set enrichment analysis using DEPICT on full sets of GWAS summary statistics of SBP, DBP, and PP resulted in 1842 significant pathways (FDR < 0.01) in total, of which 791 were shared among the three BP phenotypes (Figure 5). In line with the abovementioned results, terms related to the development and organogenesis of the cardiovascular system are amongst the most significant results (Supplementary Table S12).

#### 2.3.3. Potential Mediating Mechanisms from Sequences to Consequences

**Figure 6:**
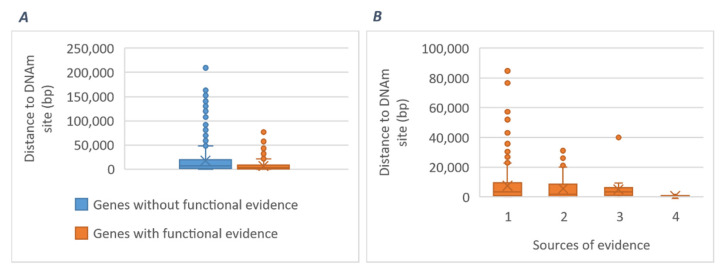
Comparison of the physical distance of 219 genes with and 613 genes without any functional evidence (**A**), as well as of 219 genes with different sources of evidence (**B**) to their nearest DNA methylation (DNAm) sites. DNAm signals are generated by the MSMR analysis (SMR analysis of GWAS vs. mQTL) and are mapped to their nearest genes based on Ensemble GRCh37 release 98.

SMR analyses of the three BP traits using mQTL data by McRae et al. [38] returned 658, 693, and 583 DNA methylation (DNAm) sites for SBP, DBP, and PP, respectively (Bonferroni-corrected P_SMR < 5.56 × 10^−7^; P_HEIDI ≥ 0.01). A total of 1176 identified DNAm sites were novel for BP. All significant DNAm sites jointly map to 832 genes, of which 219 were supported by at least one source of functional evidence from our post-GWAS analyses. Comparison of the physical distance of 219 genes with and 613 genes without any functional evidence to their nearest DNAm sites suggests DNAm sites closer to genes are more likely to be causal (Figure 6).

SMR analysis of mQTL versus eQTL resulted in 11,378 DNAm sites significantly associated with expression levels of 6253 genes, which were not rejected by the HEIDI test (Bonferroni-corrected P_SMR < 1.85 × 10^−8^; P_HEIDI ≥ 0.01). The latter analysis was used for mapping BP-associated methylation levels to BP-associated gene expressions and enabled us to find 80 genes for which the BP-associated gene expression was mediated by methylation (Supplementary Table S13).

The gene expression association of these 80 genes with BP is mediated by 106 DNAm sites. For many genes, multiple DNAm sites serve as regulatory mediators, of which *ACADVL*, *FBXW2*, and *UTP11L* with seven, seven, and six DNAm sites are the outstanding examples.

### 2.4. Converging Evidence for the Most Highly Prioritised Genes

To inform the choice of the most promising genes for future studies, we listed essential characteristics of the most highly prioritised genes based on the current work in Table 1. The table includes 15 genes with significant evidence from at least four out of six different prioritization approaches, i.e., coding consequences, multilayer molecular pleiotropic associations, MX, SMR, DEPICT, and 3× SMR (Figure 1). These genes were then followed up in the top six relevant tissues for gene expression associations with BP levels (Table 1). Apart from three genes which were not available in GTEx TWAS results, eleven out of the twelve highly prioritised genes were successfully replicated in cardiovascular tissues.

Our analyses also detected 34 likely causal genes with more than one source of biological evidence, which were not previously linked to BP. These genes, as well as the 15 highly prioritised genes, were selected for replication.

### 2.5. Replication of the Prioritised Genes in an Independent Population

All the 15 most highly prioritised genes were replicated in the Million Veteran Program (MVP) cohort (*p*-value < 0.05). For the newly identified genes, 28 out of 34 (~82%) were replicated (*p*-value < 0.05) (Table 2, Supplementary Table S14).

## 3. Discussion

We performed several integrative analyses to translate GWAS results of human blood pressure into biological insights. We aimed to detect the most likely causal genes underlying the control of BP and validated these genes in an independent population. We also depicted how gene expression associations with BP traits vary among different tissues and showed the most relevant tissues. Finally, we shed light on the major BP-regulating mechanisms which the prioritised genes act through. Our results highlight prioritised genes, relevant tissues, biological pathways, and possible drug targets for BP that can be followed up to be translated into clinical practice.

By prioritizing a total of 1880 biologically plausible genes for BP, we were able to shed light on the functional relevance of BP GWAS loci. We integrated genomics, epigenomics, and transcriptomics of BP and were the first to link these omics levels through a likely causal pathway chain using large-scale data in blood with follow-up analyses in six cardiovascular tissues. Adding to the results of multi-omics studies in kidneys [22], the percentage of BP genomic loci with likely causal genes now reaches ~85%. Of note, about half of the prioritised genes would not have been identified by merely mapping 901 BP loci to their nearest genes. Conversely, only 496 out of the 901 nearest genes (~55%) were supported by any source of biological evidence from our extensive number of different bioinformatics analyses. This is in line with previous evidence [39] confirming that genes nearest to the most significant GWAS SNPs are not always the most likely to be functional.

### 3.1. Highly Prioritzed Genes and New Genes

We provided a list of the most highly prioritised genes supported by more than three lines of evidence for further translational research. A few examples are *TRIOBP* and *USP36* which show consistent associations with lower BP levels in almost all relevant tissues and *MAK16* and *ERAP2* which are found to have consistent associations with higher BP levels (Table 1).

Nine out of the 15 highly prioritised genes have not yet been functionally investigated for BP, i.e., *TRIOBP*, *USP36*, *LRIG1*, *PRR14*, *MLXIP*, *YAF2*, *SLC39A1*, *MAPKAP1*, and *MAK16* (Table 1), and should be considered for further follow-up studies. *TRIOBP* encodes for a cytoskeleton remodelling protein which binds to F-actin [40], and its variants are associated with intraocular pressure [41]. Cytoskeleton remodelling is the mediating mechanism through which cardiovascular cells can sense and transfer the mechanical environment signals into the nucleus to modify cell behavior [42]. Our functional predictions showed its potential involvement in abnormal vascular endothelial cell morphology. *USP36*, encoding a ubiquitin-specific peptidase, is a deubiquitination enzyme which has been shown to decrease ischemic injury in renal tubular cells [43]. Functional estimates showed its likely involvement in the *FLG2* protein–protein interaction subnetwork which maintains epithelial homeostasis and barrier function [44]. *LRIG1* encodes for a transmembrane protein, with its variants associated with carotid intima media thickness [45] and atrial fibrillation [46]. In line with our enrichment analyses showing *LRIG1* among the epidermal growth factor and fibroblast growth factor signalling pathways, it is proposed to be involved in proliferation regulation of epidermal stem cells [47]. *PRR14*, encoding a proline-rich protein, is involved in myoblast differentiation and morphogenesis [48]. *MLXIP*, which encodes the MLX interacting protein, regulates pathways involved in myocyte glucose uptake [49]. *YAF2*, encoding YY1-associated factor 2, is mostly expressed in heart and skeletal muscle [50] and is involved in skeletal and cardiac muscle cells differentiation [51]. *SLC39A1* encodes a solute carrier which is mapped to the epidermal differentiation complex [52] and may also act as a cell–ECM junction based on DEPICT coregulation analyses. *MAPKAP1*, encoding mitogen-activated protein kinase-associated protein 1, also known as *SIN1*, has the highest expression in heart and skeletal muscle [53] and, in line with our functional investigations, is a component of TORC2, a protein kinase complex involved in AKT phosphorylation and cell signalling [54]. It is targeted by the currently developed drugs and could be considered for repurposing. *MAK16* encodes for an RNA-binding motif protein, the homologous protein of which has been implicated in 60S ribosome biogenesis in *Saccharomyces cerevisiae* [55].

We further identified several new genes for BP, of which 34 were supported by more than one source of biological evidence. Twenty-eight out of the 34 genes (~82%) were successfully replicated in the MVP cohort (*n* = ~220,000) (Table 2).

Two of the novel genes, i.e., *RPS6KB1* and *MAP1A*, show interactions with a number of available drugs, of which metaraminol has the primary indication for hypotension (Supplementary Table S9a). Other interacting drugs can be considered as opportunities for drug repositioning.

Six other novel genes are indicated as druggable by DGIdb. An example is growth factor receptor-bound protein 2-related adaptor protein (*GRAP*) which is involved in the Ras signalling pathway. The exact function is not known, but its homolog gene in *Drosophila* is suggested to play a role in regulating the actin cytoskeleton dynamics system [56]. Based on our coregulation analysis using DEPICT, this gene is predicted to be involved in abnormal vascular endothelial cell development, with its expression being among the top genes in endothelial cells. *GRAP* gene expression showed a positive association with DBP in both TWAS approaches (Supplementary Tables S4b and S5b). Another example is signal peptidase complex subunit 1 (*SPCS1*) with an nsSNP linked to BP loci (r^2^ > 0.5). *SPCS1* gene expression is positively associated with SBP and PP (Supplementary Table S5a,c). It is also among the genes with evidence of mediation by DNA methylation (Supplementary Table S13). It has been shown that this gene has a key role in posttranslational modification of some structural proteins [57]. *FAM212A*, also known as *INKA1* (inka box actin regulator 1), is another druggable target with significant evidence of negative association with DBP at both the DNA methylation and gene expression levels (Supplementary Table S13). It serves as an inhibitor of PAK4 [58], which is involved in cytoskeleton remodelling [59].

Novel genes also include those that encode regulatory RNAs such as long noncoding RNAs (lncRNAs) and small nuclear RNAs (snRNAs). The biological importance of regulatory RNAs in complex traits/diseases is getting more and more attention [60,61,62]. An example of a novel lncRNA gene is *RP11-10A14.3* with its expression being consistently associated with an increase in all the three BP traits and its methylation being consistently associated with a decrease in all the three BP traits. An example of a novel snRNA gene is *RNU6-510P* (Supplementary Table S13). Similarly to *RP11-10A14.3* but in an inverse direction, *RNU6-510P* gene expression is negatively associated with SBP and PP, and its methylation is positively associated with both BP traits (Supplementary Table S13). Further studies are worthwhile to elucidate their exact mechanisms in regulating BP.

### 3.2. Mechanistic Insights

#### 3.2.1. Gene-Specific Mechanisms

Here, we describe mechanistic details of a few BP genes with a focus on regulatory mechanisms through DNA methylation and gene expression. For this goal, the results of 3× SMR analyses were followed up with the Roadmap Epigenomics Mapping Consortium (REMC) [36] and Encyclopedia of DNA Elements (ENCODE) project [37] data to annotate the regulatory features of the 3× SMR significant loci and mediating methylation sites. For a few genes, we found further biological evidence supporting the complete causal chain from DNA to BP.

**Figure 7:**
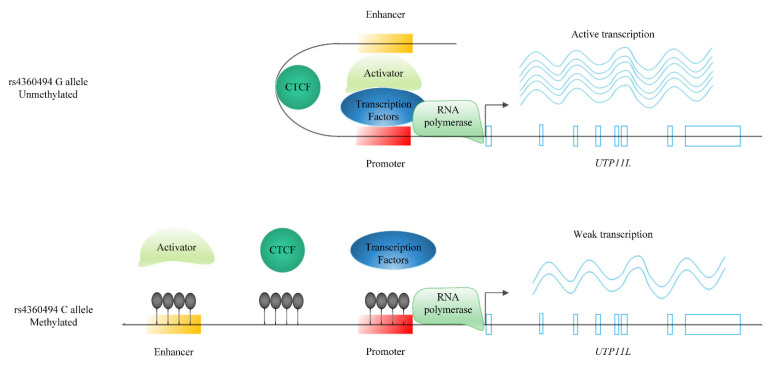
Potential regulatory mechanism for *UTP11L* through which a BP-associated variant controls BP-associated gene expression. SNP rs4360494 is positively correlated with the expression of *UTP11L*. At the same time, this SNP is inversely associated with the methylation level of three sites at the promoter and the early sequence of this gene as well as the upstream enhancer and an active CTCF-binding site. The overall hypothesis is that rs4360494 regulates gene expression by controlling the methylation of the promoter and the enhancer as well as the CTCF-binding site. CTCF increases the expression of *UTP11L* in the presence of the rs4360494 G allele by involving enhancer activity through DNA remodelling, and disruption of CTCF binding due to the methylated DNA in the presence of the rs4360494 C allele decreases the expression of the *UTP11L* gene.

One example is *UTP11L.* This gene encodes the U3 small nucleolar ribonucleoprotein which is expressed highly in muscles [63,64], and its expression level is negatively correlated with SBP (β_SMR_ = −0.91) (Supplementary Table S5a). One of the 901 lead SNPs for BP, rs4360494 is positively correlated with the expression of *UTP11L* (β_SMR_ = 0.24) (Supplementary Table S5a). At the same time, this SNP is inversely associated with the methylation level of three sites (cg27018070, cg01941663, and cg26528311) at the promoter and the early sequence of this gene as well as the upstream enhancers [65] (Supplementary Figure S1). SMR results also confirm the inverse association of these methylation sites with the expression level of *UTP11L* (β_SMR_ = −0.67, −0.91, and −0.92 for the three probes, respectively) (Supplementary Table S13). The plausible mechanism by which rs4360494 affects the expression level of the *UTP11L* gene would be via controlling the methylation of its promoter and enhancer. Besides, cg27018070 colocalises with an active CTCF binding site in myotubes [65]. CTCF has shown a potential role in regulating gene expression via remodelling the chromatin structure [66]. A more complete hypothesis is that CTCF increases the expression of *UTP11L* in the presence of the rs4360494 G allele by involving enhancer activity through DNA remodelling. On the other hand, the disruption of CTCF binding to the methylated DNA in the presence of the rs4360494 C allele decreases the expression of the *UTP11L* gene (Figure 7). The slightly different directions of effects among three BP traits may give a clue to diverse biological mechanisms regulating different BP traits. *UTP11L* and *FHL3*, another SMR significant gene upstream, together with their top related genes are enriched in myoblast differentiation (FDR < 0.1) [34]. *UTP11L* is a druggable target gene (Supplementary Table S9d) and could be considered for BP therapeutics.

Two other interesting examples are *PPL* and *ITGA9,* which, in addition to SMR analyses, were also prioritised based on coding consequences, MX analysis, and DEPICT.

*PPL* encodes periplakin with membranous expression in squamous epithelium [64] and, according to our SMR analyses, its expression is positively correlated with BP (Supplementary Tables S5a–c and S12). Our 3× SMR analyses showed that its expression is regulated through methylation of the downstream region of the gene. According to the REMC project data [36], this region is mapped to the downstream promoter and epithelial enhancer of the gene (Supplementary Figure S2). Interestingly, both GeneMANIA and DEPICT enrichment analyses predicted the function of *PPL* to be related to epithelial development and differentiation and cell–extracellular matrix junctions.

*ITGA9* encoding integrin alpha 9 is also mostly expressed in squamous epithelium [64]. Our SMR results show its expression to be positively correlated with BP (Supplementary Tables S5a–c and S12). Besides, Supplementary Table S13 shows that the expression of *ITGA9* is positively regulated by the methylation of an upstream region (Supplementary Figure S3), which maps to a transcription factor-binding site for ZBTB33 [37]. The observed increased interaction of ZBTB33 with methylated DNA can explain this association [67]. Nevertheless, the expression of these two genes i.e., *ITGA9* and *ZBTB33*, is positively correlated [68]. According to coregulation analyses using DEPICT, this gene is amongst the top ten genes of hypotension, dilated heart atrium, and abnormal epithelium morphology gene sets. In addition to the role of endothelial *ITGA9* in angiogenesis [69] and venous valve formation [70], the combination of these gene sets may suggest a new role in heart endocardium, inner layer of epithelial cells. This is concordant with the evidence of high expression of ZBTB33 in the heart [68]. *ITGA9*, together with its top related genes, is involved in extracellular matrix organization based on GeneMANIA predictions. It is a druggable target (Supplementary Table S9d) and could be considered for BP therapeutics.

Another example is the *ERAP2* gene which was also highlighted based on our multilayer association lookups (Supplementary Table S3). *ERAP2*, the nearby gene to rs709668 amongst the seven lead BP SNPs with multilayer molecular associations (Figure 2), encodes for endoplasmic reticulum aminopeptidase 2 which plays a role in angiogenesis and BP regulation [71,72] and is positively correlated with BP (Supplementary Table S5a,b). A locus within the 18th intron of the *ERAP2* gene affects the methylation of the blood enhancer region at the early sequence of the nearby gene *LNPEP* [36] (Supplementary Figure S4) with related functions (based on GeneMANIA predictions) and positive correlation with BP. The methylation of this site coincidentally increases the expression of *ERAP2* and *LNPEP* (Supplementary Table S13) which may be explained partly via suppressing a cryptic promoter yielding a higher expression [73] and/or complementarily by other potential molecular mediators. These two genes are known drug targets (Supplementary Table S9c) and could be considered for repurposing.

Two other genes with high levels of evidence from different prioritization approaches are *TP53INP1* and *SENP2*. *TP53INP1* encodes for tumour protein p53 inducible nuclear protein 1. It is associated with hypertension [74] and is involved in autophagy [75,76]. Our functional enrichment analyses suggest its contribution to kidney failure. SMR analyses show its positive correlation with BP (Supplementary Table S5a,b); 3× SMR analyses suggest that the increased methylation level of its upstream promoter decreases the expression of *TP53INP1* and, ultimately, BP (Supplementary Figure S5 and Table S13). *SENP2*, encoding SUMO specific peptidase 2, modulates the Wnt signalling pathway and in turn, heart development [77]. It is also reported to be involved in adipogenesis [78]. We showed that its expression level is positively correlated with SBP (Supplementary Table S5a). *SENP2* and *TP53INP1* are druggable targets (Supplementary Table S9d) and can be considered for drug design.

#### 3.2.2. General Mechanisms

Our functional enrichment results using all the 1880 prioritised genes suggest that amongst the four major categories mentioned as the driving regulators of BP, i.e., cardiac output, peripheral vascular resistance, humoral mediators, and the sympathetic nervous system [79], the first two play the main roles in the genetic component of BP regulation. To name a few, terms related to cardiac muscle and chamber morphogenesis, angiogenesis procedure, epithelial tube formation, and vascular smooth muscles differentiation are frequently observed among enriched functions. All these can be seen as primary determinants of the mechanical properties of blood flow and major contributors to cardiovascular haemodynamics. Furthermore, our functional enrichment results (Supplementary Tables S9–S11) highlight the involvement of the ECM, which in combination with other terms may point towards arterial stiffness [80]. There is strong evidence to support that remodelling of the small arteries in hypertension indicates a remodelling of the ECM and of the extracellular–vascular SMC attachment sites by integrins. Alterations in ECM components, integrins, and other adhesion molecules result in a rearrangement of SMCs and a restructured vascular wall [81]. Vascular muscle cell hyperplasia and accumulation of ECM proteins, compounded by vascular calcification, reduce the elasticity of large vessels and culminate in arterial stiffness [82,83], which is an independent risk factor for cardiovascular diseases and target organ damage [84]. Further supporting evidence for the involvement of arterial stiffness in BP regulation comes from the observed correlations and also shared genes for these two traits [85,86].

The expression and function of our most highly prioritised genes provide links to the vascular endothelium, vascular and cardiac muscle cells, and heart in terms of morphology and development. These findings suggest that arterial stiffness and endothelial dysfunction are key components of the pathogenesis of hypertension [82,83].

Trait-specific functional analysis revealed cardiovascular anatomical structure, kidney function, and ECM organization as specific mechanisms for regulation of systolic, diastolic, and pulse pressure, respectively.

The results of tissue prioritization which highlights arteries, muscles, and connective adipose tissues are in line with the abovementioned conclusions on the major BP regulatory mechanisms and also with previous studies [7,9,19]. In addition, the substantial pleiotropic effects of BP loci on heart rate and also associations with aortic root sise (Supplementary Table S1) provide further supporting evidence for the important role of the mechanical properties of blood flow in BP regulation.

### 3.3. Clinical Insights

We also searched drug databases for interactions with our prioritizsd genes in order to translate the results into potentially useful clinical information. The list of drugs interacting with our prioritised genes covered ~80% of all the TTD drugs [33], with the primary indication for hyper/hypotension, which provides confidence in our results. In addition, non-BP drugs targeting our prioritised genes make new candidates for repositioning. An example is Tosedostat, which shows interactions with seven prioritised genes for BP, including two of the most highly prioritised. This drug has shown hypotensive side effects in more than 30% of patients [87]. Obviously, these results require careful follow-up in complementary studies that evaluate different pharmacological aspects of suggested drug targets and suitability of each drug for repositioning. Our results can also be added to the current drug target prioritization tools [88] to help identify the most suitable drug targets. Moreover, specific mechanistic insights on the regulation of different BP traits can be of importance for developing or even prescribing appropriate treatments for patients with different BP complications.

### 3.4. Future Perspectives

Beyond DNA methylation and gene expression, there is a variety of other molecular layers that might be informative in biological translation of GWAS signals. Although we tried to leverage proteomics and metabolomics through our multilayer molecular association analysis, more comprehensive efforts, including these, as well as other molecular signatures such as other epigenetic markers and splicing effects, are the possible next steps. We also did not include rare variants (MAF < 0.01) or indels, as these were not present in the ICBP/UKB GWAS data. When available, addition of such variants should be informative and may be incorporated in future studies.

Our integrative multi-omics study is based on the largest meta-GWAS on BP to date including over 750,000 European people, with its results explaining over 10% of the total blood pressure heritability of around 50% [2,3]. Our results help to inform key components and mechanistic insights into BP regulation. Larger meta-GWAS efforts on BP are ongoing, which promise further success in explaining the genetic component of BP and can be followed by more comprehensive post-GWAS studies. Trans-ancestry analyses are also required to make the results more generalizable.

## 4. Materials and Methods

### 4.1. GWAS Data

We worked on full sets of GWAS meta-analyses results (i.e., summary statistics) from >750,000 individuals representing the discovery stage based on the combination of International Consortium of Blood Pressure (ICBP) and UK Biobank GWAS of SBP, DBP, and PP provided by Evangelou et al. [9].

### 4.2. Gene Prioritization

We used large-scale blood datasets where genetic–molecular associations were needed (i.e., Section 4.2.2 and Section 4.2.3) and replicated the prioritised genes in the relevant cardiovascular tissues. We reasoned the use of this strategy in the Introduction as well as in Appendix A.

#### 4.2.1. Coding Consequences of BP Loci

We performed in silico sequencing based on the 901 GWAS SNPs (gSNPs) reported by Evangelou et al. [9] using our previously published pipeline [11] to investigate the coding consequences of BP-associated loci. We prioritised genes with nonsynonymous variations linked (r^2^ > 0.5) to 901 BP loci. We also investigated the severity of their functional consequences (Supplementary Methods).

#### 4.2.2. Seeking Pleiotropic Associations of BP Loci with Different Molecular Traits

We used Phenoscanner [28] to look up the associations of 901 gSNPs with different molecular traits in the European population, including DNA methylation, gene expression, protein levels, and metabolite levels. Herein, we call pleiotropic associations with all four molecular layers multilayer molecular associations and consider a gene to be prioritised if it has associations with all the four molecular traits.

#### 4.2.3. Transcriptome-Wide Association Study (TWAS) Using Summary Statistics

We performed TWAS analyses on blood expression datasets with large sample sizes, i.e., eQTLGen (*n* = ~32,000) [89] and DGN (*n* = 922) [90], to empower the detection of likely causal genes, using two different methods, i.e., MX and SMR to reciprocally confirm significant associations. Both methods rely on genetic data and potentially suffer from LD confounding effects due to multiple distinct but linked causal variants for gene expression and the trait. We identified and excluded possibly confounded results using appropriate tests for each TWAS approach, i.e., the COLOC [91] and HEterogeneity In Dependent Instruments (HEIDI) [14] tests for MX and SMR, respectively (Supplementary Methods).

#### 4.2.4. Determining Coregulated Genes within BP-Associated Loci

We also conducted gene prioritization analysis using DEPICT [29], which is based on functional similarities for all genes, even functionally unknown, that lie within trait-associated loci according to co-expression data. Our analyses were based on the DEPICT default settings, i.e., LD r^2^ < 0.05 and physical distance of 500 kb for clumping, with a GWAS *p*-value threshold of 5.0 × 10^−8^. We ran DEPICT on full sets of GWAS summary statistics of SBP, DBP, and PP separately.

### 4.3. Tissue Prioritization

Aiming to identify the most relevant context for the prioritised genes to be followed, we repeated TWAS analyses, including both MX and SMR, but used 48 tissues from the GTEx consortium [92], based on the three GWAS of SBP, DBP, and PP. We prioritised tissues based on their enrichment of gene expression associations, measured by the average squares of the Z-scores calculated for the association of BP traits and genetic components of gene expression levels. Since GTEx v7 lacked other important tissues like kidney, we also performed DEPICT tissue enrichment analysis which utilises a set of 37,427 human microarray samples to identify tissue/cell types in which genes from the associated loci are highly expressed. Highly relevant tissues were then used to confirm gene expression associations of the most highly prioritised genes with BP levels.

### 4.4. Functional Follow-Up of the Genetically Prioritised Genes

We merged the prioritised gene sets for each BP trait separately and for all the BP traits combined by including genes with at least one source of biological evidence, i.e., genes (i) with coding consequences, (ii) from multilayer associations with molecular traits, (iii) with expression levels associated with BP, (iv) coregulated with other genes from BP-associated loci. Then, we used the final merged lists of prioritised genes for downstream analyses (Figure 1). We also selected the most highly prioritised genes by scoring genes based on the number of different prioritization methods providing evidence for that gene. After that, we reviewed literature for the investigated functions of those genes in relation to BP using ISI Web of Science with the following search strategy based on the topic of study (TS) and using ALL languages and ALL document types across ALL timespans:

TS = (Gene_name AND (“blood pressure” OR hyp*tension))

#### 4.4.1. Suitability of Prioritised Genes as Drug Targets

To investigate the clinical relevance of our prioritised genes, we queried the DGIdb [31] for known drug–gene interactions or druggable categories. Next, we investigated if the interacting drugs are primarily indicated for hyper-/hypotension using the TTD [33]. We also checked if the interacting drugs have BP-related adverse effects using the SIDER side effect resource [32]. Finally, we examined the enrichment of BP drug targets among our list of prioritised genes.

#### 4.4.2. Functional Enrichment Analyses of Prioritised Genes

To better understand the functional consequences of our prioritised genes (see above) for each BP trait as well as for all the BP traits combined, we used them as input to construct composite networks followed by functional enrichment analysis. We applied Cytoscape v3.7.1 extended by the GeneMania plugin v3.4.1 using its comprehensive set of accompanying databases. We used the RamiGO R package [93] for the visualization of significant GO terms within the appropriate GO tree. As a negative control, we constructed five random gene sets with the same number as our prioritised gene list and repeated the abovementioned steps.

Additionally, another set of enrichment analyses was completed using DEPICT (see above) with its 14,461 reconstituted gene sets based on co-expression data of 77,840 samples.

#### 4.4.3. Potential Mediating Mechanisms: From Sequences to Consequences

To further investigate the potential mediating mechanisms from DNA to BP, we ran two extra rounds of SMR analyses, first on the blood mQTL data versus three BP GWASs (SBP, DBP, and PP), and second on blood mQTL data versus blood eQTL data. Then, we added the TWAS results (see above) and determined the intersection of all the three runs as robust evidence for mediatory effects of epigenetic mechanisms in BP regulation (i.e., DNA → methylation → gene expression → BP). For details, see Supplementary Methods (3× SMR section) and the study by Wu et al. [13].

### 4.5. Replication of the Prioritised Genes in an Independent Population

We employed both TWAS approaches (i.e., MX and SMR) using BP GWAS results of the MVP cohort (*n* = 220,680) from the Giri et al. study [19] to independently confirm our most highly prioritised genes as well as the newly identified genes with more than one source of evidence.

## 5. Conclusions

To conclude, recent advances in GWAS discovery of BP have shed light on the hitherto hidden side of BP biology, i.e., its genetic component. Here, we tried to translate BP GWAS results into biological insights and provided the likely causal genes that should be targeted in the most relevant tissues alongside with the suggested mechanisms involved. We prioritised hundreds of likely causal genes, among which we identified and successfully validated 28 new genes for BP. Our examination of gene expression–BP associations in different tissues prioritised arteries, muscles, subcutaneous adipose tissue, and fibroblasts. Mechanisms of BP genetic regulation were mostly related to cardiovascular structures, with anatomical structures more specific for SBP, kidney development more specific for DBP, and extracellular matrix organization more specific for PP. Follow-up of these results may eventually yield more precise, individualised control of BP.

## Figures and Tables

**Table 1:** The most highly prioritised genes with evidence from at least four different prioritization methods suggested for further follow-up studies.

Gene	Evidence	nsSNP	Effect Direction in the Prioritised Tissues ^a^		Likely Causal in Kidneys ^b^	Reported ^c^/Predicted ^d,e^ Mechanism(s)	Druggability	Drug Name
TRIOBP *	NS, MX, SMR, DEPICT	rs12628603	**↓↓↓↓↓↓**	(SBP, PP) ^†^		Abnormal vascular endothelial cell morphology		
USP36 *	NS, MX, SMR, DEPICT	rs3088040	**↓↓**−−**↓↓**	(SBP, DBP, PP) ^†^	×	FLG2 PPI subnetwork	#	
LRIG1 *	NS, MX, SMR, DEPICT	rs2306272	−**↓**−**↓**−	(SBP, PP) ^†^		EGF, FGF, and ERBB signalling pathways		
PRR14 *	NS, MX, SMR, DEPICT	rs3747481	−−−−−−	(DBP, PP)		KAT2A PPI subnetwork		
*SENP2*	NS, MX, SMR, DEPICT	rs6762208	**↑↑**−−−−	(SBP, DBP) ^†^		Wnt signalling pathway	#	
*PPL*	NS, MX, SMR, DEPICT, 3× SMR	rs1049205	−−−−−−	(SBP, DBP, PP)		Epithelium development, likely mediated by methylation		
MLXIP *	NS, MX, SMR, DEPICT	rs7978353	−−−−**↓**−	(SBP, DBP) ^†^		FGF signalling pathway		
*ITGA9*	NS, MX, SMR, DEPICT, 3× SMR	rs267561	−−−−−−	(SBP, DBP, PP)		Dilated heart atrium and abnormal epithelium morphology, likely mediated by methylation	#	
YAF2 *	MX, SMR, DEPICT, 3× SMR		**↓↓↑**−**↓**−	(DBP) ^†^		Chromatin modification, likely mediated by methylation	#	
*LNPEP*	MX, SMR, DEPICT, 3× SMR		**↑**−−**↑↑**− **↑**−−↓↓−	(SBP, DBP) ^†^ (PP) ^†^		Hemopoietic or lymphoid organ development, likely mediated by methylation	¥	TOSEDOSTAT
SLC39A1 *	MX, SMR, DEPICT, 3× SMR		−−↓−−−	(DBP)		Cell–matrix adhesion, likely mediated by methylation	#	
MAPKAP1 *	MX, SMR, DEPICT, 3× SMR		−**↓**−−**↓**−	(SBP, DBP, PP) ^†^	×	EGF, FGF, and ERBB signalling pathways, likely mediated by methylation	¥	INK-128, OSI-027, AZD-8055
*TP53INP1*	MX, SMR, DEPICT, 3× SMR		−−−−−↓ −−−−−↑	(SBP) (DBP)	×	Increased cell proliferation, increased urine protein level, kidney failure, likely mediated by methylation	#	
MAK16 *	NS, MX, SMR, 3× SMR	rs6468171	−**↑↑↑↑**−	(PP) ^†^		Ribosomal biogenesis, likely mediated by methylation		
*ERAP2*	ML, MX, SMR, 3× SMR		**↑↑↑↑↑↑**	(SBP, DBP) ^†^		Angiogenesis, likely mediated by methylation.	¥	TOSEDOSTAT

* Genes with an asterisk have not yet been functionally studied for BP regulation based on an ISI Web of Science search TS (i.e., topic) = (Gene_name AND (“blood pressure” OR hyp*tension)). ^a^ From TWAS analyses using GTEx eQTL data; the arrows show the direction of effect for the association of gene expression and BP traits in the prioritised tissues, including tibial artery, aorta, skeletal muscle, subcutaneous adipose tissue, fibroblasts, and heart left ventricle, using MetaXcan and SMR analyses, respectively. TWAS associations with the BP trait of evidence for each gene are shown, and the trait of evidence is mentioned next to them. † Nominally significant associations for at least one of the traits of evidence, with consistent direction of effect across both TWAS approaches in any of the prioritised tissues. Dashes mean the gene data are not available in the GTEx dataset(s). ^b^ From the study by Eales et al. [22]. ^c^ From literature. ^d^ From DEPICT or GeneMania enrichment analyses. ^e^ The mediating mechanism by which the gene is regulated; from 3× SMR analyses of GWAS, eQTL, and mQTL data. Evidence: any biological evidence from our post-GWAS analyses; nsSNP, nonsynonymous SNP; NS, genes with nsSNP linked (r^2^ > 0.5) to GWAS loci; ML, multilayer molecular associations; MX, MetaXcan; SMR, summary-based Mendelian randomization. ¥ indicates that the gene is targeted by (a) known drug(s); # indicates that the gene is druggable according to the DGI database.

**Table 2:** Summary of the 28 newly identified genes and their replication in the MVP cohort using TWAS analysis.

Gene Name	No. of Evidence	Trait(s) of Evidence	min_P_rep	Druggability	Drug Name(s)
*SPCS1*	3	SBP, PP	2.39 × 10^−2^	#	
*GRAP*	3	DBP	5.35 × 10^−3^	#	
*NT5DC2*	3	PP	1.62 × 10^−2^		
*FAM212A*	3	SBP, DBP	1.07 × 10^−2^	#	
*RPS6KB1*	2	SBP, PP	4.61 × 10^−7^	¥	METARAMINOL *, LY-2584702, LY-2780301, XL-418, BETHANIDINE SULFATE, SIROLIMUS, MSC-2363318A
*HOXA7*	2	SBP, DBP, PP	2.03 × 10^−2^	#	
*NIP7*	2	DBP	7.13 × 10^−3^		
*CCDC97*	2	DBP, PP	4.42 × 10^−4^		
*CENPV*	2	DBP	1.43 × 10^−2^	#	
*MAP1A*	2	DBP	2.03 × 10^−3^	¥	ESTRAMUSTINE
*TTC16*	2	SBP	5.00 × 10^−4^		
*LTBP3*	2	SBP, PP	1.70 × 10^−2^	#	
*NSG2*	2	SBP, DBP	5.09 × 10^−5^		
*RP5-874C20.3*	2	DBP, PP	1.55 × 10^−8^		
*HIST1H2BH*	2	SBP, DBP	5.48 × 10^−3^		
*TPM2*	2	DBP	1.75 × 10^−2^		
*RNU6-510P*	2	SBP, PP	1.45 × 10^−10^		
*AC116366.6*	2	SBP, DBP	2.33 × 10^−2^		
*RP11-464F9.1*	2	SBP, DBP, PP	7.30 × 10^−4^		
*RP11-464F9.9*	2	SBP, DBP, PP	9.45 × 10^−4^		
*RNU6-415P*	2	PP	5.32 × 10^−4^		
*RP11-10A14.3*	2	SBP, DBP, PP	3.21 × 10^−7^		
*RP11-148O21.4*	2	SBP, DBP	1.95 × 10^−4^		
*RP11-59H1.3*	2	SBP, PP	1.07 × 10^−2^		
*RP3-473L9.4*	2	SBP, DBP, PP	2.00 × 10^−5^		
*ZNF234*	2	SBP, DBP	4.04 × 10^−2^		
*AF131215.9*	2	SBP, DBP, PP	8.34 × 10^−6^		
*RP11-148O21.6*	2	SBP, DBP	1.35 × 10^−3^		

SBP, systolic blood pressure; DBP, diastolic blood pressure; PP, pulse pressure; min_P_rep, minimum *p*-value of replication. ¥ indicates that the gene is targeted by (a) known drug(s); # indicates that the gene is druggable according to the DGI database. * METARAMINOL is a known drug with the primary indication for hypotension.

**Figure A1:**
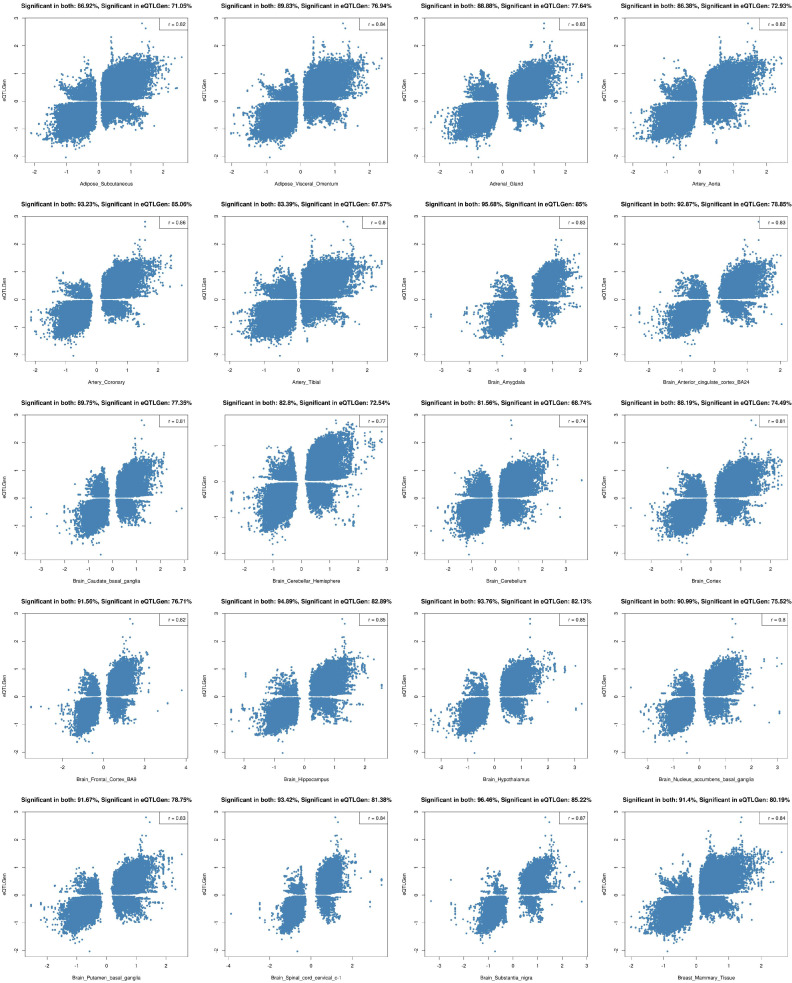

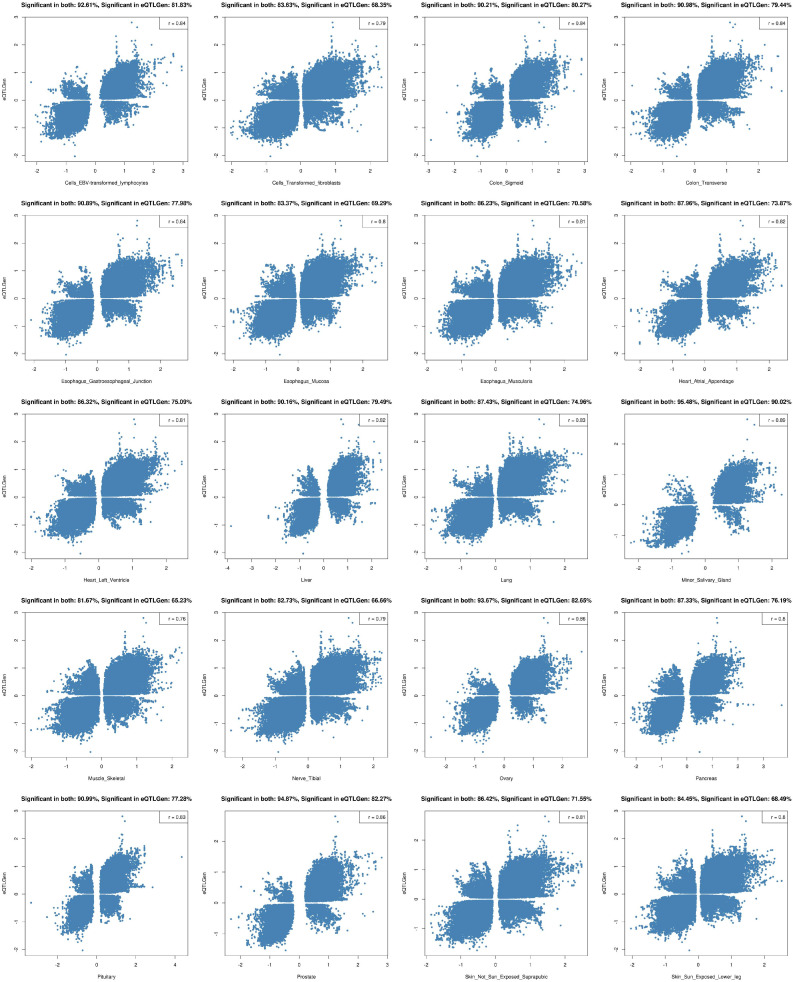

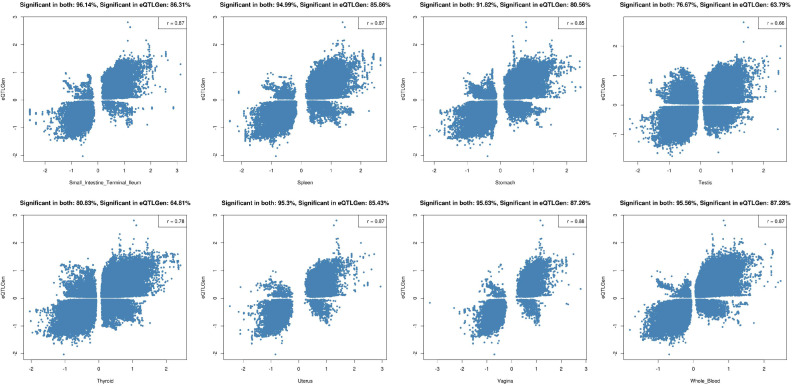
Correlation of effect estimates between eQTLGen dataset and significant (FDR < 0.05) eQTLs from 48 GTEx tissues. Two numbers are reported above each plot: ‘Significant in both’ indicates the percentage of genome-wide significant (GW-sig) eQTLs in each tissue that are also GW-sig in eQTLGen; ‘Significant in eQTLGen’ indicates the percentage of FDR-significant eQTLs of each tissue that that reach GW-sig only in eQTLGen. Palindromic SNPs were removed prior to comparison.

## Data Availability

The BP datasets analysed for this study can be found in the GWAS catalog (https://www.ebi.ac.uk/gwas/, accessed on 15 September 2018) ftp repository. The analysed mQTL summary data are available at SMR website (https://yanglab.westlake.edu.cn/software/smr/, accessed on 18 February 2019). The eQTL data analysed are available at the eQTLGen (https://www.eqtlgen.org/, accessed on 12 April 2018) and PredictDB (https://predictdb.org/, accessed on 8 January 2019) websites. All the results generated for this study can be found in the article/Supplementary Materials.

## Supplementary Materials

The following supporting information can be downloaded at: https://www.mdpi.com/article/10.3390/ijms23147557/s1. References [94,95,96] are cited in the supplementary materials.

## Appendix A

Previous comprehensive studies on eQTLs showed that proximal genetic regulatory effects (also called with prefix ‘cis’) are mostly non-tissue-specific [24,89]. Here, we investigated the overlap of eQTLs from the large eQTLGen blood dataset with 48 GTEx tissues to evaluate the extent to which analyses in blood are representative of cis-regulatory mechanisms in different tissues (Figure A1).

As shown in the figure, the majority of FDR-significant eQTLs (FDR < 0.05) in different GTEx datasets with tiny sample sizes are present in the eQTLGen dataset. Moreover, many of the FDR-significant eQTLs from GTEx datasets that do not pass the genome-wide significant *p*-value threshold pass this threshold in eQTLGen. As SMR is based on highly significant eQTLs (*p* < 5 × 10^−8^), this constitutes an additive value for using eQTLGen by providing more power to detect regulatory associations. We did not test the correlation of mQTL data from a variety of tissues with blood. However, Qi et al. already showed that the correlation of other tissue datasets with blood for mQTLs is even stronger than for eQTLs [24]. There might be still a minority of tissue-specific effects which remains elusive. Meanwhile, pinpointing true tissue-specific associations from dataset-specific false positives is challenging in the absence of replication datasets and small sample sizes for individual tissues. Moreover, there is no evidence of the enrichment of tissue-specific eQTLs for associations with complex traits [97].
