# Supplementary material for: Large-Scale Multi-Omics Studies Provide New Insights into Blood Pressure Regulation
Source: Int J Mol Sci. 2022 Jul 8;23(14):7557. doi: 10.3390/ijms23147557 (PMC9323755; doi:10.3390/ijms23147557)
Supplement: Supplementary file 1 [file ijms-23-07557-s001.zip › ijms-1782779-supplementary_final.pdf]

DATA SUPPLEMENT

Large-scale multi-omics studies provide new insights into blood pressure regulation

Zoha Kamali [1,2], Jacob M. Keaton [3,4], Shaghayegh Haghjooy Javanmard [5,6], International Consortium of Blood Pressure, Million Veteran Program, eQTLGen consortium, BIOS consortium, Todd L. Edwards [7], Harold Snieder [1,†] and Ahmad Vaez [1,2,*,†]

[1] Department of Epidemiology, University of Groningen, University Medical Centre Groningen, 9713 GZ Groningen, The Netherlands; z.kamali@umcg.nl (Z.K.); h.snieder@umcg.nl (H.S.)
[2] Department of Bioinformatics, Isfahan University of Medical Sciences, Isfahan. P.O. Box 81746-7346, Iran
[3] Division of Epidemiology, Department of Medicine, Vanderbilt University Medical Center, Nashville, TN. 37203, USA; jacob.keaton@vumc.org
[4] Center for Precision Health Research, National Human Genome Research Institute, National Institutes of Health, Bethesda, MD. 20894, USA
[5] Applied Physiology Research Centre, Cardiovascular Research Institute, Isfahan University of Medical Sciences, Isfahan. P.O. Box 81746-7346, Iran; shaghayegh.haghjoo@gmail.com
[6] Regenerative Medicine Research Center, Isfahan University of Medical Sciences, Isfahan. P.O. Box 81746-7346, Iran
[7] Division of Epidemiology, Department of Medicine, Vanderbilt Genetics Institute, Vanderbilt University Medical Center, Nashville, TN. 37204, USA; todd.l.edwards@vumc.org
* Correspondence: a.vaez@umcg.nl
† These authors have contributed equally to this work.

Contents

Supplementary Methods 3
In silico sequencing pipeline 3
Transciptome-wide association studies (TWASs) 3
Tissue prioritization 4
3xSMR analysis 4
Supplementary Figures 6
Supplementary Figure S1. Results of 3xSMR analyses for *UTP11L* gene 6
Supplementary Figure S2. Results of 3xSMR analyses for *PPL* gene 7
Supplementary Figure S3. Results of 3xSMR analyses for *ITGA9* gene. 8
Supplementary Figure S4. Results of 3xSMR analyses for *ERAP2* gene. 9
Supplementary Figure S5. Results of 3xSMR analyses for *TP53INP1* gene. 10
Consortia information 12
ICBP (International Consortium of Blood Pressure) 12
MVP (Million Veteran Program) 17
eQTLGen 21
BIOS (Biobank-based Integrative Omics Study) 22
References 24

Supplementary Methods

In silico sequencing pipeline

Our locus definition was based on r^2> 0.5 as linkage disequilibrium (LD) metric and a physical distance of 1 Mb on both sides of the lead SNP. For LD calculations we used PLINK v1.9 (1) based on the 1000 Genomes Project phase 3 (2) data, including only subjects of European ancestry. For variant annotations, we used ANNOVAR (3) (version 16 April 2018). We then used Ensembl variant effect predictor (VEP) (4) release 95 to identify gene names of BP linked non-synonymous SNPs (nsSNPs) and also to investigate the severity of their functional consequences indexed by SIFT and Polyphen scores.

Transciptome-wide association studies (TWASs)

In order to identify genes where expression levels are associated with BP free of non-genetic confounders, we performed MX analysis for systolic-, diastolic-, and pulse pressure GWASs, separately, using gene expression prediction models from whole blood Depression Genes and Networks (DGN) data (5). We only included GWAS variants with minor allele frequency (MAF) ≥0.01 and excluded all SNPs and genes within the MHC region. According to 11,430 genes being tested in DGN analysis, a Bonferroni corrected significance level of $<4.37\times10^{-06}$ was applied. We then excluded genes with prediction performance p-values larger than Bonferroni corrected thresholds i.e. $0.05/n_{sign}$ (where n_{sign} is the number of MX significant genes for BP) as suggested by Barbeira *et al*. (6).

To address the LD contamination concern in our MX significant genes, we also tested colocalization of association signals for BP and gene expression using the COLOC R package (7). The COLOC test is typically based on approximate Bayes factors on five hypotheses of 1) no causal variant (H0), 2) causal variant for BP only (H1), 3) causal variant for gene expression only (H2), 4) two distinct causal variants (H3) and 5) one common causal variant (H4) for both traits.

We performed COLOC analysis on SBP, DBP, and PP GWASs separately using the eQTLGen whole blood eQTL dataset. This analysis was based on all SNPs in common between GWAS and eQTL datasets and no filtering was applied based on p-values. We took a probability of H3<0.5 and H4>0.5 as acceptable evidence of colocalized signals to filter out MX TWAS association results, which can be due to LD, as suggested by Barbeira *et al* (6).

Further to investigate gene expression associations with BP using MR causal inference, we ran SMR analysis (8) on the three BP GWASs (SBP, DBP and PP). We also conducted the HEIDI test to exclude LD confounded results.

We used the blood cis-eQTL summary data from the eQTLGen consortium (n ~32,000) (9). The 1000 Genomes Project phase3 data of European population was used for LD calculations through SMR (8). We only included GWAS variants with MAF≥0.01 and excluded those variants with either inconsistent alleles or MAF differences >0.20 amongst pairs of the three datasets,

i.e., GWAS, eQTL, and 1000 Genomes datasets. We also excluded all variants and expression probes within the MHC region.

Considering 15,368 genes being tested, we used a Bonferroni corrected significance level of $<3.25\times10^{-06}$ for SMR. We selected a p-value threshold of ≥0.01 for HEIDI as acceptable evidence of not being confounded by LD, which is more stringent than a Bonferroni corrected significance level.

Tissue prioritization

We repeated TWAS analyses using both MX and SMR methods over 48 GTEx datasets to find tissues in which gene expression associations with BP traits are stronger. For each tissue, we first calculated average squared Z-scores (the below formula) of genes against 3 BP traits and two TWAS methods (MX and SMR), i.e. six calculations per tissue.

$$Z_t^{(p)} = \frac{\sum_{i=1}^{n} Z_i^2}{n}$$

With t being the tissue, p the BP trait, i each individual gene, and n the total number of tested genes. Next, we normalized these $Z_t^{(p)}$ values for each TWAS method and against each BP outcome, using the mean and standard deviation of $Z_t^{(p)}$ values across tissues, to get harmonized comparable measures. Finally, we calculated for each tissue, the average of the six normalized $Z_t^{(p)}$ values as a measure of tissue priority for BP traits.

3xSMR analysis

We ran SMR analyses on the three BP GWASs (SBP, DBP and PP) using blood mQTL summary data. We used the mQTL results of a meta-analysis of the Brisbane Systems Genetics Study and Lothian Birth Cohorts (combined n = 1,980) reported by McRae *et al* (10).

We assessed the novelty of DNA methylation (DNAm) probes according to the Epigenome-Wide Association Studies (EWAS) catalog and the recently published multi-omics study of BP in kidney (11,12). We also investigated whether the physical distance of DNAm probes to their nearest genes is deterministic in the functionality of those genes, by comparing the physical distance to DNAm sites between genes with and without biological evidence from our pipeline, as well as among genes with different lines of evidence.

Then for mapping methylation probes to their likely affected genes, we repeated SMR analysis on the mQTL data, described above, using eQTL data from the eQTLGen consortium (9). We only included GWAS variants with MAF≥0.01 and excluded all variant and methylation probes within the MHC region. We applied appropriate Bonferroni corrected significance thresholds for SMR tests and a stringent threshold of ≥0.01 for the HEIDI tests. LD reference data and settings for all HEIDI tests is the same as previously (see TWAS methods). Finally, merging the three sets

of SMR analyses (3xSMR) including GWAS vs. mQTL (MSMR), mQTL vs. eQTL (MESMR) and GWAS vs. eQTL (ESMR), enabled us to link DNA to BP through methylation mediated gene expression at specific sites (i.e., DNA→Methylation→Gene expression→BP).

Afterwards we used the Roadmap Epigenomics Mapping Consortium (REMC) (13) and Encyclopedia of DNA Elements (ENCODE) project (14) data based on the Ensembl regulatory build (15) to annotate the known regulatory features of the 3xSMR significant loci and mediating methylation sites.

Supplementary Figures

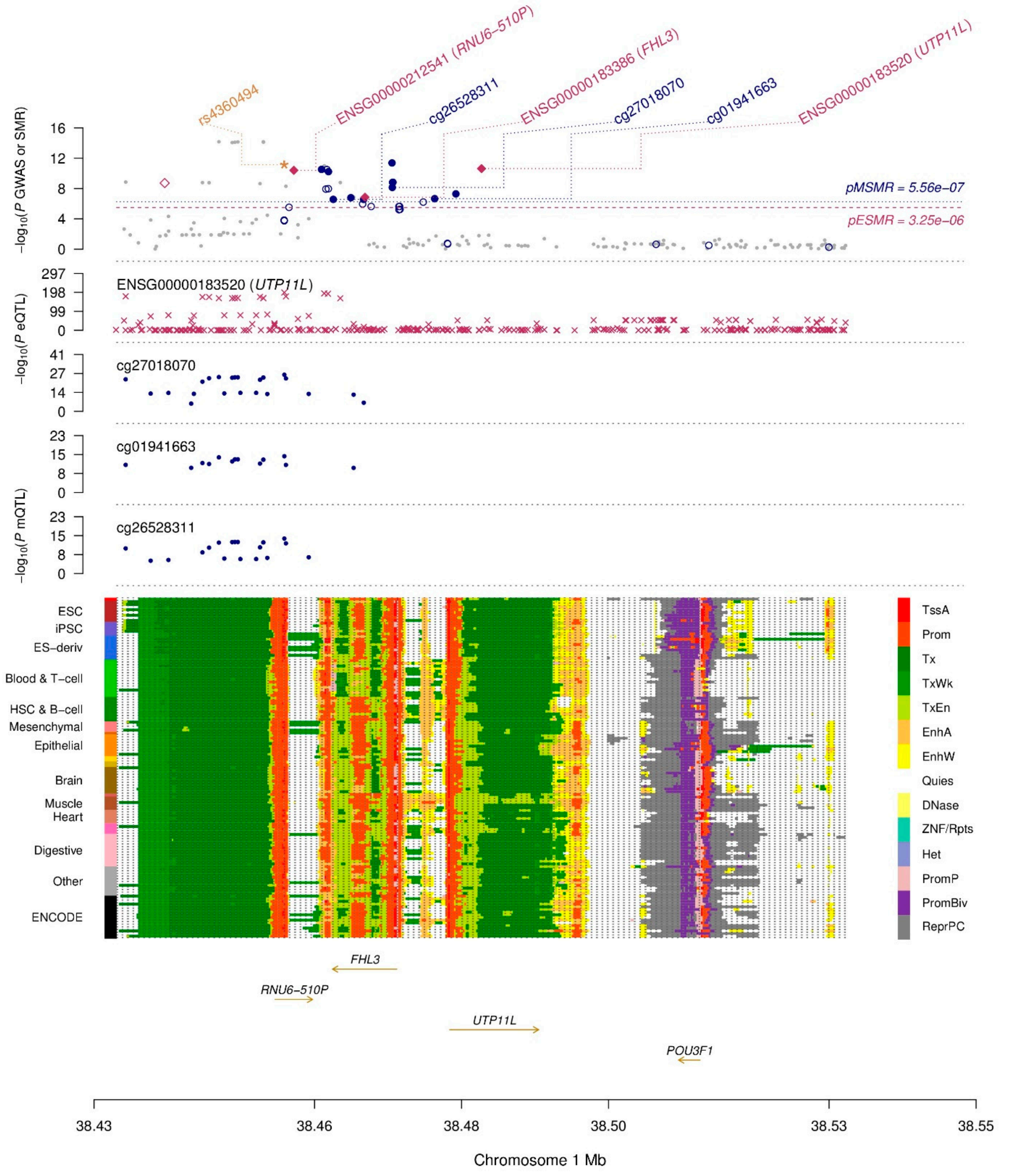

Supplementary Figure S1. Results of 3xSMR analyses for *UTP11L* gene. The top plot shows the meta-analysis −log10(P-values) of SNPs from the SBP GWAS. The red diamonds represent −log10(P-values) of SMR analysis of eQTL data (ESMR) and the blue circles represent −log10(P-values) of SMR analysis of mQTL data (MSMR). Probes which were not rejected by the HEIDI test are filled in with solid colors. The second plot shows −log10(P-values) of the association of eQTL SNPs for *UTP11L* and its expression level. The third plot shows −log10(P-values) of the associations of mQTL SNPs and

methylation probes for *UTP11L*. The bottom plot shows chromatin state annotations from REMC and ENCODE for different cells/tissue types.

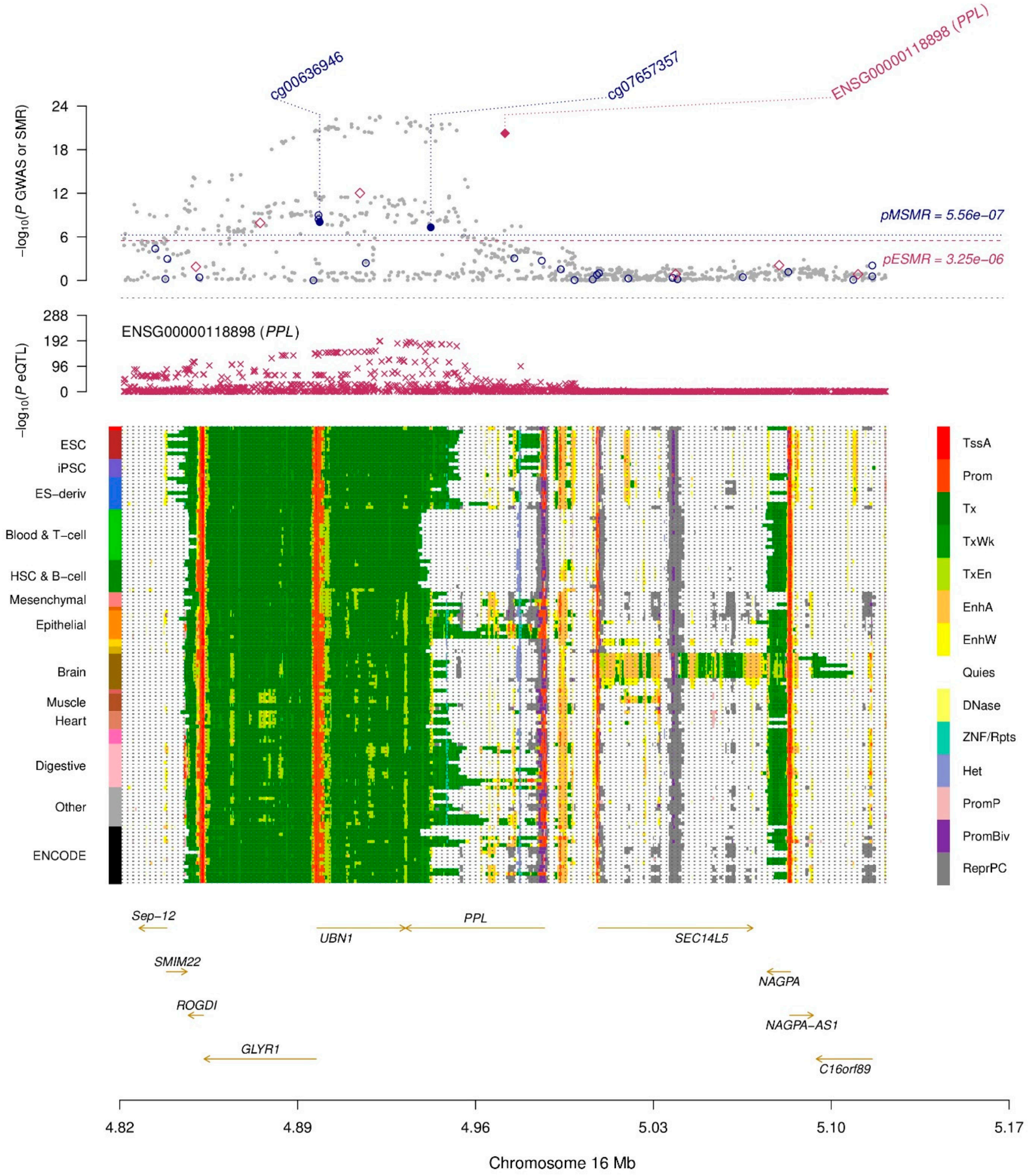

Supplementary Figure S2. Results of 3xSMR analyses for *PPL* gene.

The plot shows results of SMR analyses using mQTL, eQTL and GWAS data. The top plot shows the meta-analysis −log10(P-values) of SNPs from the GWAS results. The red diamonds represent −log10(P-values) of SMR analysis of eQTL data (ESMR) and the blue circles represent −log10(P-values) of SMR analysis of mQTL data (MSMR). Probes which were not rejected by the HEIDI test are filled in with solid

colors. The second plot shows −log10(P-values) of the association of eQTL SNPs and their corresponding gene expression levels. The bottom plot shows chromatin state annotations from REMC and ENCODE for different cells/tissue types.

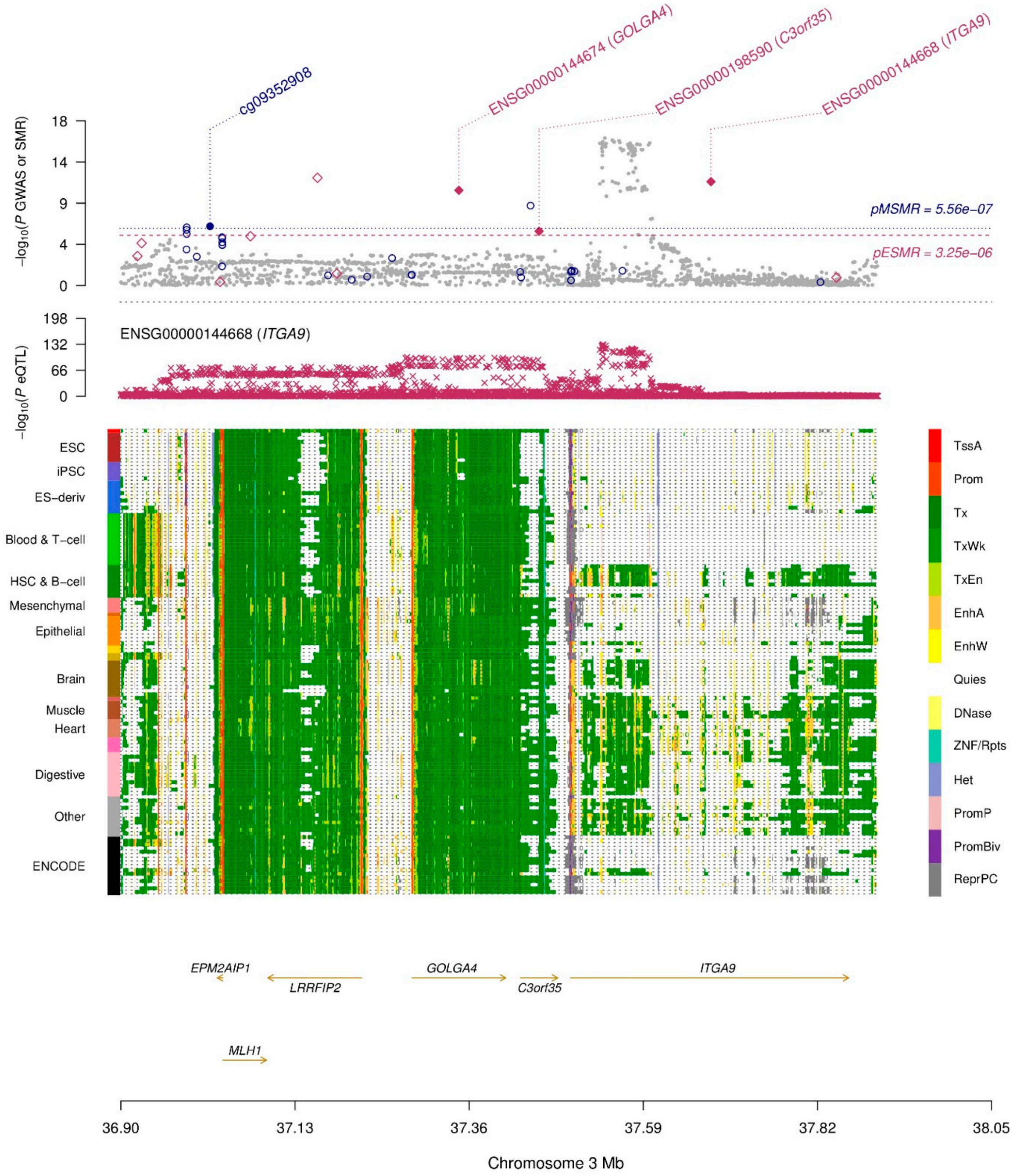

Supplementary Figure S3. Results of 3xSMR analyses for *ITGA9* gene.

The plot shows results of SMR analyses using mQTL, eQTL and GWAS data. The top plot shows the meta-analysis −log10(P-values) of SNPs from the GWAS results. The red diamonds represent −log10(P-values) of SMR analysis of eQTL data (ESMR) and the blue circles represent −log10(P-values) of SMR analysis of mQTL data (MSMR). Probes which were not rejected by the HEIDI test are filled in with solid

colors. The second plot shows −log10(P-values) of the association of eQTL SNPs and their corresponding gene expression levels. The bottom plot shows chromatin state annotations from REMC and ENCODE for different cells/tissue types.

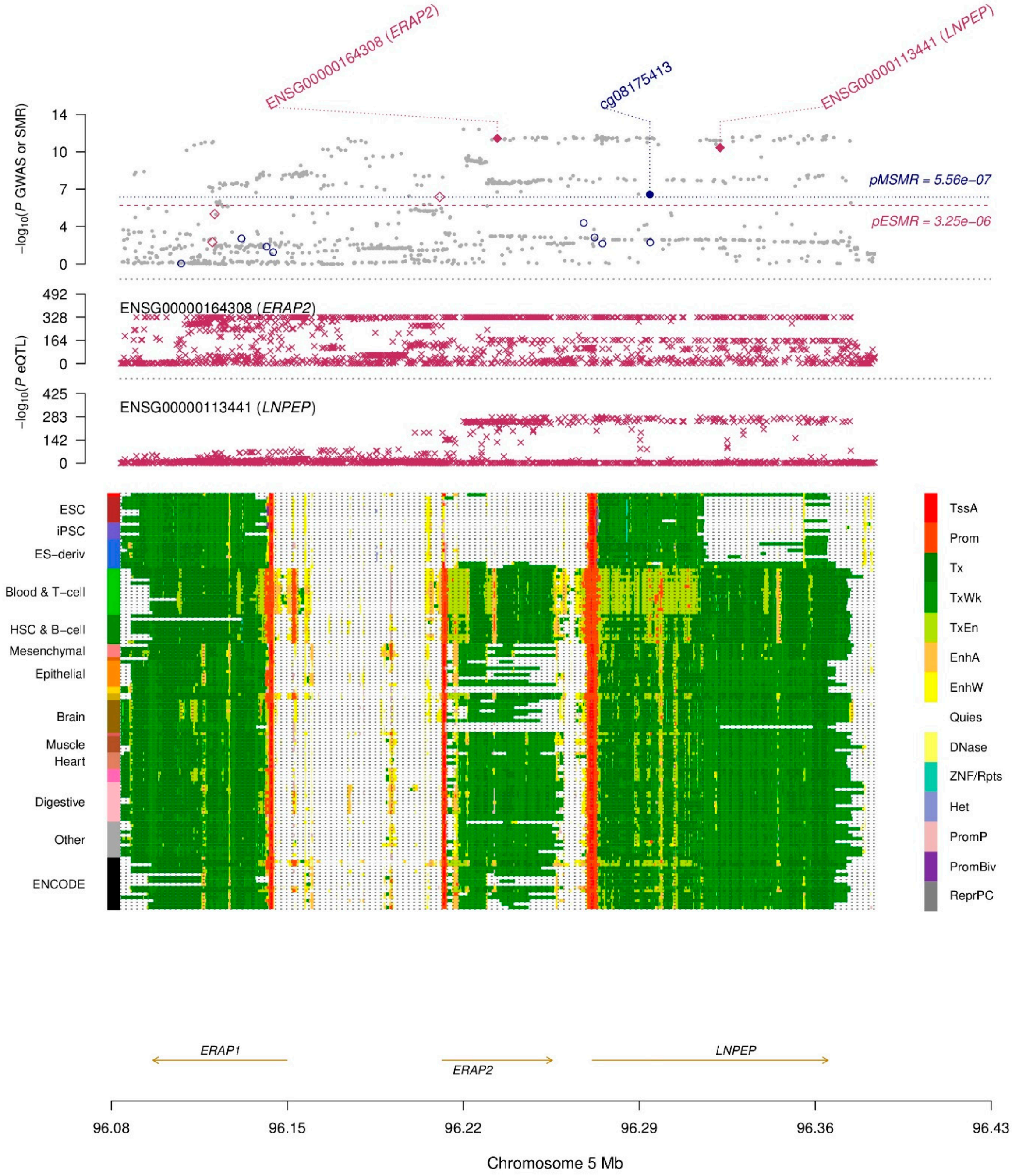

Supplementary Figure S4. Results of 3xSMR analyses for *ERAP2* gene. The plot shows results of SMR analyses using mQTL, eQTL and GWAS data. The top plot shows the meta-analysis −log10(P-values) of SNPs from the GWAS results. The red diamonds represent −log10(P-values) of SMR analysis of eQTL data (ESMR) and the blue circles represent −log10(P-values) of SMR analysis of mQTL data (MSMR). Probes which were not rejected by the HEIDI test are filled in with solid

colors. The second plot shows −log10(P-values) of the association of eQTL SNPs and their corresponding gene expression levels. The bottom plot shows chromatin state annotations from REMC and ENCODE for different cells/tissue types.

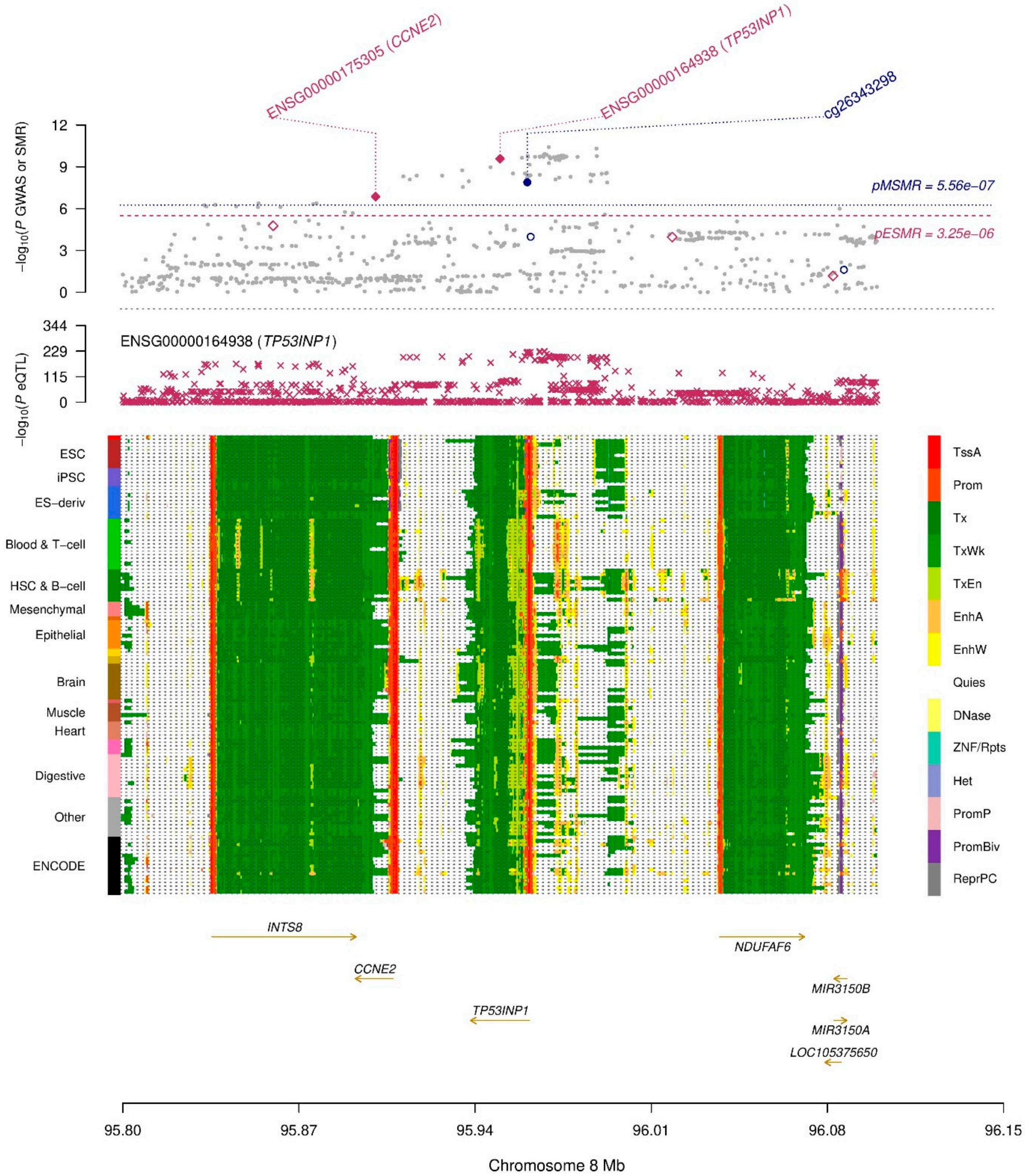

Supplementary Figure S5. Results of 3xSMR analyses for *TP53INP1* gene. The plot shows results of SMR analyses using mQTL, eQTL and GWAS data. The top plot shows the meta-analysis −log10(P-values) of SNPs from the SBP GWAS results. The red diamonds represent −log10(P-values) of SMR analysis of gene expression probes (ESMR) and the blue circles represent −log10(P-values) of SMR analysis of methylation probes (MSMR). Probes which were not rejected by the HEIDI test are filled in with solid colors. The second plot shows −log10(P-values) of the association of eQTL SNPs and their

corresponding gene expression levels. The bottom plot shows chromatin state annotations from REMC and ENCODE for different cells/tissue types

Consortia information

ICBP (International Consortium of Blood Pressure)

Evangelos Evangelou[1,2], Helen R Warren[3,4], He Gao[1,5], Georgios Ntritsos[2], Niki Dimou[2], Tonu Esko[16,17], Reedik Mägi[16], Lili Milani[16], Peter Almgren[18], Thibaud Boutin[19], Stéphanie Debette[20,21], Jun Ding[22], Franco Giulianini[23], Elizabeth G Holliday[24], Anne U Jackson[25], Ruifang Li-Gao[26], Wei-Yu Lin[27], Jian'an Luan[28], Massimo Mangino[29,30], Christopher Oldmeadow[24], Bram Peter Prins[31], Yong Qian[22], Muralidharan Sargurupremraj[21], Nabi Shah[32,33], Praveen Surendran[27], Sébastien Thériault[34,35], Niek Verweij[17,36,37], Sara M Willems[28], Jing-Hua Zhao[28], Philippe Amouyel[38], John Connell[39], Renée de Mutsert[26], Alex SF Doney[32], Martin Farrall[40,41], Cristina Menni[29], Andrew D Morris[42], Raymond Noordam[43], Guillaume Paré[34], Neil R Poulter[44], Denis C Shields[45], Alice Stanton[46], Simon Thom[47], Gonçalo Abecasis[48], Najaf Amin[49], Dan E Arking[50], Kristin L Ayers[51,52], Caterina M Barbieri[53], Chiara Batini[54], Joshua C Bis[55], Tineka Blake[54], Murielle Bochud[56], Michael Boehnke[25], Eric Boerwinkle[57], Dorret I Boomsma[58], Erwin P Bottinger[59], Peter S Braund[60,61], Marco Brumat[62], Archie Campbell[63,64], Harry Campbell[65], Aravinda Chakravarti[50], John C Chambers[1,5,66-68], Ganesh Chauhan[69], Marina Ciullo[70,71], Massimiliano Cocca[72], Francis Collins[73], Heather J Cordell[51], Gail Davies[74,75], Martin H de Borst[76], Eco J de Geus[58], Ian J Deary[74,75], Joris Deelen[77], Fabiola Del Greco M[78], Cumhur Yusuf Demirkale[79], Marcus Dörr[80,81], Georg B Ehret[50,82], Roberto Elosua[83,84], Stefan Enroth[85], A Mesut Erzurumluoglu[54], Teresa Ferreira[86,87], Mattias Frånberg[88-90], Oscar H Franco[91], Ilaria Gandin[62], Paolo Gasparini[62,72], Vilmantas Giedraitis[92], Christian Gieger[93-95], Giorgia Girotto[62,72], Anuj Goel[40,41], Alan J Gow[74,96], Vilmundur Gudnason[97,98], Xiuqing Guo[99], Ulf Gyllensten[85], Anders Hamsten[88,89], Tamara B Harris[100], Sarah E Harris[63,74], Catharina A Hartman[101], Aki S Havulinna[102,103], Andrew A Hicks[78], Edith Hofer[104,105], Albert Hofman[91,106], Jouke-Jan Hottenga[58], Jennifer E Huffman[19,107,108], Shih-Jen Hwang[107,108], Erik Ingelsson[109,110], Alan James[111,112], Rick Jansen[113], Marjo-Riitta Jarvelin[1,5,114-116], Roby Joehanes[107,117], Åsa Johansson[85], Andrew D Johnson[107,118], Peter K Joshi[65], Pekka Jousilahti[102], J Wouter Jukema[119], Antti Jula[102], Mika Kähönen[120,121], Sekar Kathiresan[17,36,122], Bernard D Keavney[123,124], Kay-Tee Khaw[125], Paul Knekt[102], Joanne Knight[126], Ivana Kolcic[127], Jaspal S Kooner[5,67,68,128], Seppo Koskinen[102], Kati Kristiansson[102], Zoltan Kutalik[56,129], Maris Laan[130], Marty Larson[107], Lenore J Launer[100], Benjamin Lehne[1], Terho Lehtimäki[131,132], David CM Liewald[74,75], Li Lin[82], Lars Lind[133], Cecilia M Lindgren[40,87,134], YongMei Liu[135], Ruth JF Loos[28,59,136], Lorna M Lopez[74,137,138], Yingchang Lu[59], Leo-Pekka Lyytikäinen[131,132], Anubha Mahajan[40], Chrysovalanto Mamasoula[139], Jaume Marrugat[83], Jonathan Marten[19], Yuri Milaneschi[140], Anna Morgan[62], Andrew P Morris[40,141], Alanna C Morrison[142], Peter J Munson[79], Mike A Nalls[143,144], Priyanka Nandakumar[50], Christopher P Nelson[60,61], Teemu Niiranen[102,145], Ilja M Nolte[146], Teresa Nutile[70], Albertine J Oldehinkel[147], Ben A Oostra[49], Paul F O'Reilly[148], Elin Org[16], Sandosh Padmanabhan[64,149], Walter Palmas[150], Aarno Palotie[103,151,152], Alison Pattie[75], Brenda WJH Penninx[140], Markus Perola[102,103,153], Annette Peters[94,95,154], Ozren Polasek[127,155], Peter P Pramstaller[78,156,157], Quang Tri Nguyen[79], Olli T Raitakari[158,159], Rainer Rettig[161], Kenneth Rice[162], Paul M Ridker[23,163], Janina S Ried[94], Harriëtte Riese[147], Samuli Ripatti[103,164], Antonietta Robino[72], Lynda M Rose[23], Jerome I Rotter[99], Igor Rudan[165], Daniela Ruggiero[70,71], Yasaman Saba[166], Cinzia

F Sala[53], Veikko Salomaa[102], Nilesh J Samani[60,61], Antti-Pekka Sarin[103], Reinhold Schmidt[104], Helena Schmidt[166], Nick Shrine[54], David Siscovick[167], Albert V Smith[97,98], Harold Snieder[146], Siim Sõber[130], Rossella Sorice[70], John M Starr[74,168], David J Stott[169], David P Strachan[170], Rona J Strawbridge[88,89], Johan Sundström[133], Morris A Swertz[171], Kent D Taylor[99], Alexander Teumer[81,172], Martin D Tobin[54], Maciej Tomaszewski[123,124], Daniela Toniolo[53], Michela Traglia[53], Stella Trompet[119,173], Jaakko Tuomilehto[174-177], Christophe Tzourio[21], André G Uitterlinden[91,178], Ahmad Vaez[146,179], Peter J van der Most[146], Cornelia M van Duijn[49], Germaine C Verwoert[91], Veronique Vitart[19], Uwe Völker[81,180], Peter Vollenweider[181], Dragana Vuckovic[62,182], Hugh Watkins[40,41], Sarah H Wild[183], Gonneke Willemsen[58], James F Wilson[19,65], Alan F Wright[19], Jie Yao[99], Tatijana Zemunik[184], Weihua Zhang[1,67], John R Attia[24], Adam S Butterworth[27,185], Daniel I Chasman[23,163], David Conen[186,187], Francesco Cucca[188,189], John Danesh[27,185], Caroline Hayward[19], Joanna MM Howson[27], Markku Laakso[190], Edward G Lakatta[191], Claudia Langenberg[28], Olle Melander[18], Dennis O Mook-Kanamori[26,192], Colin NA Palmer[32], Lorenz Risch[193-195], Robert A Scott[28], Rodney J Scott[24], Peter Sever[128], Tim D Spector[29], Pim van der Harst[196], Nicholas J Wareham[28], Eleftheria Zeggini[31], Daniel Levy[107,118], Patricia B Munroe[3,4], Christopher Newton-Cheh[134,197,198], Morris J Brown[3,4], Andres Metspalu[16], Bruce M. Psaty[201,202], Louise V Wain[54], Paul Elliott[1,5,203-205], Mark J Caulfield[3,4]

1. Department of Epidemiology and Biostatistics, Imperial College London, London, UK.
2. Department of Hygiene and Epidemiology, University of Ioannina Medical School, Ioannina, Greece.
3. William Harvey Research Institute, Barts and The London School of Medicine and Dentistry, Queen Mary University of London, London, UK.
4. National Institute for Health Research, Barts Cardiovascular Biomedical Research Center, Queen Mary University of London, London, UK.
5. MRC-PHE Centre for Environment and Health, Imperial College London, London, UK.
7. Division of Epidemiology, Department of Medicine, Institute for Medicine and Public Health, Vanderbilt Genetics Institute, Vanderbilt University Medical Center, Tennessee Valley Healthcare System (626)/Vanderbilt University, Nashville, TN, USA.
8. Vanderbilt Genetics Institute, Vanderbilt Epidemiology Center, Department of Obstetrics and Gynecology, Vanderbilt University Medical Center; Tennessee Valley Health Systems VA, Nashville, TN, USA.
9. Department of Epidemiology, Emory University Rollins School of Public Health, Atlanta, GA, USA.
10. Department of Biomedical Informatics, Emory University School of Medicine, Atlanta, GA, USA.
11. Massachusetts Veterans Epidemiology Research and Information Center (MAVERIC), VA Boston Healthcare System, Boston, USA.
12. Division of Aging, Department of Medicine, Brigham and Women's Hospital, Boston, MA, Department of Medicine, Harvard Medical School, Boston, MA, USA.
13. Atlanta VAMC and Emory Clinical Cardiovascular Research Institute, Atlanta, GA, USA.
14. VA Palo Alto Health Care System; Division of Cardiovascular Medicine, Stanford University School of Medicine, CA, USA.
15. Nephrology Section, Memphis VA Medical Center and University of Tennessee Health Science Center, Memphis, TN, USA.
16. Estonian Genome Center, University of Tartu, Tartu, Estonia.
17. Program in Medical and Population Genetics, Broad Institute of Harvard and MIT, Cambridge, MA, USA.
18. Department Clinical Sciences, Malmö, Lund University, Malmö, Sweden.
19. MRC Human Genetics Unit, MRC Institute of Genetics and Molecular Medicine, University of Edinburgh, Western General Hospital, Edinburgh, Scotland, UK
20. Department of Neurology, Bordeaux University Hospital, Bordeaux, France.
21. Univ. Bordeaux, Inserm, Bordeaux Population Health Research Center, CHU Bordeaux, Bordeaux, France.
22. Laboratory of Genetics and Genomics, NIA/NIH , Baltimore, MD, USA.
23. Division of Preventive Medicine, Brigham and Women's Hospital, Boston, MA, USA.
24. Hunter Medical Reseach Institute and Faculty of Health, University of Newcastle, New Lambton Heights, New South Wales, Australia.

25. Department of Biostatistics and Center for Statistical Genetics, University of Michigan, Ann Arbor, MI, USA.
26. Department of Clinical Epidemiology, Leiden University Medical Center, Leiden, the Netherlands.
27. MRC/BHF Cardiovascular Epidemiology Unit, Department of Public Health and Primary Care, University of Cambridge, Cambridge, UK.
28. MRC Epidemiology Unit, University of Cambridge School of Clinical Medicine, Cambridge, UK.
29. Department of Twin Research and Genetic Epidemiology, Kings College London, London, UK.
30. NIHR Biomedical Research Centre at Guy's and St Thomas' Foundation Trust, London, UK.
31. Wellcome Trust Sanger Institute, Hinxton, UK.
32. Division of Molecular and Clinical Medicine, School of Medicine, University of Dundee, UK.
33. Department of Pharmacy, COMSATS Institute of Information Technology, Abbottabad, Pakistan.
34. Department of Pathology and Molecular Medicine, McMaster University, Hamilton, Canada.
35. Institut universitaire de cardiologie et de pneumologie de Québec-Université Laval, , Quebec City, Canada.
36. Cardiovascular Research Center and Center for Human Genetic Research, Massachusetts General Hospital, Boston, Massachusetts, MA, USA.
37. University of Groningen, University Medical Center Groningen, Department of Cardiology, Groningen, The Netherlands.
38. University of Lille, Inserm, Centre Hosp. Univ Lille, Institut Pasteur de Lille, UMR1167 - RID-AGE - Risk factors and molecular determinants of aging-related diseases, Epidemiology and Public Health Department, Lille, France.
39. University of Dundee, Ninewells Hospital & Medical School, Dundee, , UK.
40. Wellcome Trust Centre for Human Genetics, University of Oxford, Oxford, UK.
41. Division of Cardiovascular Medicine, Radcliffe Department of Medicine, University of Oxford, Oxford, UK.
42. Usher Institute of Population Health Sciences and Informatics, University of Edinburgh, UK.
43. Department of Internal Medicine, Section Gerontology and Geriatrics, Leiden University Medical Center, Leiden, The Netherlands.
44. Imperial Clinical Trials Unit, Stadium House, 68 Wood Lane, London, UK.
45. School of Medicine, University College Dublin, Ireland.
46. Molecular and Cellular Therapeutics, Royal College of Surgeons in Ireland, Dublin, Ireland.
47. International Centre for Circulatory Health, Imperial College London, London, UK.
48. Center for Statistical Genetics, Dept. of Biostatistics, SPH II, Washington Heights, Ann Arbor, MI, USA.
49. Genetic Epidemiology Unit, Department of Epidemiology, Erasmus MC, Rotterdam, the Netherlands.
50. Center for Complex Disease Genomics, McKusick-Nathans Institute of Genetic Medicine, Johns Hopkins University School of Medicine, Baltimore, MD, USA.
51. Institute of Genetic Medicine, Newcastle University, Newcastle upon Tyne, UK.
52. Sema4, a Mount Sinai venture, Stamford, CT, USA.
53. Division of Genetics and Cell Biology, San Raffaele Scientific Institute, Milano, Italy.
54. Department of Health Sciences, University of Leicester, Leicester, UK.
55. Cardiovascular Health Research Unit, Department of Medicine, University of Washington, Seattle, WA, USA.
56. Institute of Social and Preventive Medicine, University Hospital of Lausanne, Lausanne, Switzerland.
57. Human Genetics Center, School of Public Health, The University of Texas Health Science Center at Houston and Human Genome Sequencing Center, Baylor College of Medicine, One Baylor Plaza, Houston, TX, USA.
58. Department of Biological Psychology, Vrije Universiteit Amsterdam, EMGO+ institute, VU University medical center, Amsterdam, the Netherlands.
59. The Charles Bronfman Institute for Personalized Medicine, Icachn School of Medicine at Mount Sinai, NY, USA.
60. Department of Cardiovascular Sciences, University of Leicester, Leicester, UK.
61. NIHR Leicester Biomedical Research Centre, Glenfield Hospital, Groby Road, Leicester, UK.
62. Department of Medical, Surgical and Health Sciences, University of Trieste, , Trieste, Italy.
63. Medical Genetics Section, Centre for Genomic and Experimental Medicine, Institute of Genetics and Molecular Medicine, University of Edinburgh, Edinburgh, UK.
64. Generation Scotland, Centre for Genomic and Experimental Medicine, University of Edinburgh, Edinburgh, UK.
65. Centre for Global Health Research, Usher Institute of Population Health Sciences and Informatics, University of Edinburgh, Edinburgh, Scotland, UK
66. Lee Kong Chian School of Medicine, Nanyang Technological University, Singapore, Singapore.
67. Department of Cardiology, Ealing Hospital, Middlesex, UK.
68. Imperial College Healthcare NHS Trust, London, UK.
69. Centre for Brain Research, Indian Institute of Science, Bangalore, India.
70. Institute of Genetics and Biophysics "A. Buzzati-Traverso", CNR, Napoli, Italy.
71. IRCCS Neuromed, Pozzilli, Isernia, Italy.
72. Institute for Maternal and Child Health IRCCS Burlo Garofolo, Trieste, Italy.
73. Medical Genomics and Metabolic Genetics Branch, National Human Genome Research Institute, NIH, Bethesda, MD, USA.

74. Centre for Cognitive Ageing and Cognitive Epidemiology, University of Edinburgh, 7 George Square, Edinburgh, UK.
75. Department of Psychology, University of Edinburgh, 7 George Square, Edinburgh, UK.
76. Department of Internal Medicine, Division of Nephrology, University of Groningen, University Medical Center Groningen, Groningen, The Netherlands.
77. Department of Molecular Epidemiology, Leiden University Medical Center, Leiden, the Netherlands.
78. Institute for Biomedicine, Eurac Research, Bolzano, Italy - Affiliated Institute of the University of Lübeck, Lübeck, Germany.
79. Mathematical and Statistical Computing Laboratory, Office of Intramural Research, Center for Information Technology, National Institutes of Health, Bethesda, MD, USA.
80. Department of Internal Medicine B, University Medicine Greifswald, Greifswald, Germany.
81. DZHK (German Centre for Cardiovascular Research), partner site Greifswald, Greifswald, Germany.
82. Cardiology, Department of Medicine, Geneva University Hospital, Geneva, Switzerland.
83. CIBERCV & Cardiovascular Epidemiology and Genetics, IMIM. Dr Aiguader 88, Barcelona, Spain.
84. Faculty of Medicine, Universitat de Vic-Central de Catalunya, Vic, Spain.
85. Department of Immunology, Genetics and Pathology, Uppsala Universitet, Science for Life Laboratory, Uppsala, Sweden.
86. Wellcome Centre for Human Genetics, University of Oxford, Roosevelt Drive, Oxford, UK.
87. Big Data Institute, Li Ka Shing Center for Health for Health Information and Discovery, Oxford University, Old Road, Oxford, UK.
88. Cardiovascular Medicine Unit, Department of Medicine Solna, Karolinska Institutet, Stockholm, Sweden.
89. Centre for Molecular Medicine, L8:03, Karolinska Universitetsjukhuset, Solna, Sweden.
90. Department of Numerical Analysis and Computer Science, Stockholm University, Stockholm, Sweden.
91. Department of Epidemiology, Erasmus MC, Rotterdam, the Netherlands.
92. Department of Public Health and Caring Sciences, Geriatrics, Uppsala, Sweden.
93. Research Unit of Molecular Epidemiology, Helmholtz Zentrum München, German Research Center for Environmental Health, Neuherberg, Germany.
94. Institute of Epidemiology, Helmholtz Zentrum München, German Research Center for Environmental Health, Neuherberg, Germany.
95. German Center for Diabetes Research (DZD e.V.), Neuherberg, Germany.
96. Department of Psychology, School of Social Sciences, Heriot-Watt University, Edinburgh, UK.
97. Faculty of Medicine, University of Iceland, Reykjavik, Iceland.
98. Icelandic Heart Association, Kopavogur, Iceland.
99. The Institute for Translational Genomics and Population Sciences, Department of Pediatrics, LABioMed at Harbor-UCLA Medical Center, Torrance, CA, USA.
100. Intramural Research Program, Laboratory of Epidemiology, Demography, and Biometry, National Institute on Aging, Bethesda, MD, USA.
101. Department of Psychiatry, University of Groningen, University Medical Center Groningen, Groningen, The Netherlands.
102. Department of Public Health Solutions, National Institute for Health and Welfare (THL), Helsinki, Finland.
103. Institute for Molecular Medicine Finland (FIMM), University of Helsinki, Helsinki, Finland.
104. Clinical Division of Neurogeriatrics, Department of Neurology, Medical University of Graz, Graz, Austria.
105. Institute for Medical Informatics, Statistics and Documentation, Medical University of Graz, Graz, Austria.
106. Department of Epidemiology, Harvard T.H. Chan School of Public Health, Boston, MA, USA.
107. National Heart, Lung and Blood Institute's Framingham Heart Study, Framingham, MA, USA.
108. The Population Science Branch, Division of Intramural Research, National Heart Lung and Blood Institute national Institute of Health, Bethesda, MD, USA.
109. Department of Medical Sciences, Molecular Epidemiology and Science for Life Laboratory, Uppsala University, Uppsala, Sweden.
110. Division of Cardiovascular Medicine, Department of Medicine, Stanford University School of Medicine, Stanford, CA USA.
111. Department of Pulmonary Physiology and Sleep, Sir Charles Gairdner Hospital, Hospital Avenue, Nedlands, Australia.
112. School of Medicine and Pharmacology, University of Western Australia.
113. Department of Psychiatry, VU University Medical Center, Amsterdam Neuroscience, Amsterdam, the Netherlands.
114. Biocenter Oulu, University of Oulu, Oulu, Finland.
115. Center For Life-course Health Research, University of Oulu, Oulu Finland.
116. Unit of Primary Care, Oulu University Hospital, Oulu, Oulu, Finland.
117. Hebrew SeniorLife, Harvard Medical School, Boston, MA, USA.
118. Population Sciences Branch, National Heart, Lung and Blood Institute, National Institutes of Health, Bethesda, MD, USA.
119. Department of Cardiology, Leiden University Medical Center, Leiden, the Netherlands.
120. Department of Clinical Physiology, Tampere University Hospital, Tampere, Finland.
121. Department of Clinical Physiology, Finnish Cardiovascular Research Center - Tampere, Faculty of Medicine and Life Sciences, University of Tampere, Tampere, Finland.

122. Broad Institute of the Massachusetts Institute of Technology and Harvard University, Cambridge, MA, USA.
123. Division of Cardiovascular Sciences, Faculty of Biology, Medicine and Health, The University of Manchester, Manchester, UK.
124. Division of Medicine, Manchester University NHS Foundation Trust, Manchester Academic Health Science Centre, Manchester, UK
125. Department of Public Health and Primary Care, Institute of Public Health, University of Cambridge, Cambridge, UK.
126. Data Science Institute and Lancaster Medical School, Lancaster, UK.
127. Department of Public Health, Faculty of Medicine, University of Split, Croatia.
128. National Heart and Lung Institute, Imperial College London, London, UK.
129. Swiss Institute of Bioinformatics, Lausanne, Switzerland.
130. Institute of Biomedicine and Translational Medicine, University of Tartu, Tartu, Estonia.
131. Department of Clinical Chemistry, Fimlab Laboratories, Tampere, Finland.
132. Department of Clinical Chemistry, Finnish Cardiovascular Research Center - Tampere, Faculty of Medicine and Life Sciences, University of Tampere, Tampere, Finland
133. Department of Medical Sciences, Cardiovascular Epidemiology, Uppsala University, Uppsala, Sweden.
134. Program in Medical and Population Genetics, Broad Institute, Cambridge, MA, USA.
135. Division of Public Health Sciences, Wake Forest School of Medicine, Winston-Salem, NC, USA.
136. Mindich Child health Development Institute, The Icahn School of Medicine at Mount Sinai, New York, NY, USA.
137. Department of Psychiatry, Royal College of Surgeons in Ireland, Education and Research Centre, Beaumont Hospital, Dublin, Ireland.
138. University College Dublin, UCD Conway Institute, Centre for Proteome Research, UCD, Belfield, Dublin, Ireland.
139. Institute of Health and Society, Newcastle University, Newcastle upon Tyne, UK.
140. Department of Psychiatry, Amsterdam Public Health and Amsterdam Neuroscience, VU University Medical Center/GGZ inGeest, Amsterdam, The Netherlands.
141. Department of Biostatistics, University of Liverpool, Block F, Waterhouse Building, Liverpool, UK.
142. Department of Epidemiology, Human Genetics and Environmental Sciences, School of Public Health, University of Texas Health Science Center at Houston, Houston, TX, USA.
143. Data Tecnica International, Glen Echo, MD, USA.
144. Laboratory of Neurogenetics, National Institute on Aging, Bethesda, USA.
145. Department of Medicine, Turku University Hospital and University of Turku, Finland.
146. Department of Epidemiology, University of Groningen, University Medical Center Groningen, Groningen, The Netherlands.
147. Interdisciplinary Center Psychopathology and Emotion regulation (ICPE), University of Groningen, University Medical Center Groningen, Groningen, The Netherlands.
148. SGDP Centre, Institute of Psychiatry, Psychology and Neuroscience, King's College London, London, UK.
149. British Heart Foundation Glasgow Cardiovascular Research Centre, Institute of Cardiovascular and Medical Sciences, College of Medical, Veterinary and Life Sciences, University of Glasgow, Glasgow, UK.
150. Department of Medicine, Columbia University Medical Center, New York, NY, USA.
151. Analytic and Translational Genetics Unit, Department of Medicine, Department of Neurology and Department of Psychiatry Massachusetts General Hospital, Boston, MA, USA.
152. The Stanley Center for Psychiatric Research and Program in Medical and Population Genetics, The Broad Institute of MIT and Harvard, Cambridge, MA, USA.
153. University of Tartu, Tartu, Estonia.
154. German Center for Cardiovascular Disease Research (DZHK), partner site Munich, Neuherberg, Germany.
155. Psychiatric hospital "Sveti Ivan", Zagreb, Croatia.
156. Department of Neurology, General Central Hospital, Bolzano, Italy.
157. Department of Neurology, University of Lübeck, Lübeck, Germany.
158. Department of Clinical Physiology and Nuclear Medicine, Turku University Hospital, Turku, Finland.
159. Research Centre of Applied and Preventive Cardiovascular Medicine, University of Turku, Turku, Finland.
161. Institute of Physiology, University Medicine Greifswald, Karlsburg, Germany.
162. Department of Biostatistics University of Washington, Seattle, WA, USA.
163. Harvard Medical School, Boston MA.
164. Public health, Faculty of Medicine, University of Helsinki, Finland
165. Centre for Global Health Research, Usher Institute of Population Health Sciences and Informatics, University of Edinburgh, Scotland, UK.
166. Gottfried Schatz Research Center for Cell Signaling, Metabolism & Aging, Molecular Biology and Biochemistry, Medical University of Graz, Graz, Austria.
167. The New York Academy of Medicine, New York, NY, USA.
168. Alzheimer Scotland Dementia Research Centre, University of Edinburgh, Edinburgh, UK.
169. Institute of Cardiovascular and Medical Sciences, Faculty of Medicine, University of Glasgow, United Kingdom.

170. Population Health Research Institute, St George's, University of London, London, UK.
171. Department of Genetics, University of Groningen, University Medical Center Groningen, Groningen, The Netherlands.
172. Institute for Community Medicine, University Medicine Greifswald, Greifswald, Germany.
173. Department of Gerontology and Geriatrics, Leiden University Medical Center, Leiden, the Netherlands.
174. Dasman Diabetes Institute, Dasman, Kuwait.
175. Chronic Disease Prevention Unit, National Institute for Health and Welfare, Helsinki, Finland.
176. Department of Public Health, University of Helsinki, Helsinki, Finland.
177. Saudi Diabetes Research Group, King Abdulaziz University, Jeddah, Saudi Arabia.
178. Department of Internal Medicine, Erasmus MC, Rotterdam, the Netherlands.
179. Research Institute for Primordial Prevention of Non-communicable Disease, Isfahan University of Medical Sciences, Isfahan, Iran.
180. Interfaculty Institute for Genetics and Functional Genomics, University Medicine Greifswald, Greifswald, Germany.
181. Department of Internal Medicine, University Hospital, CHUV, Lausanne, Switzerland.
182. Experimental Genetics Division, Sidra Medical and Research Center, Doha, Qatar.
183. Centre for Population Health Sciences, Usher Institute of Population Health Sciences and Informatics, University of Edinburgh, Scotland, UK
184. Department of Biology, Faculty of Medicine, University of Split, Croatia.
185. The National Institute for Health Research Blood and Transplant Research Unit in Donor Health and Genomics, University of Cambridge, UK.
186. Division of Cardiology, University Hospital, Basel, Switzerland.
187. Division of Cardiology, Department of Medicine, McMaster University, Hamilton, Canada.
188. Institute of Genetic and Biomedical Research, National Research Council (CNR), Monserrato, Cagliari, Italy.
189. Department of Biomedical Sciences, University of Sassari, Sassari, Italy.
190. Institute of Clinical Medicine, Internal Medicine, University of Eastern Finland and Kuopio University Hospital, Kuopio, Finland.
191. Laboratory of Cardiovascular Science, NIA/NIH , Baltimore, MD, USA.
192. Department of Public Health and Primary Care, Leiden University Medical Center, Leiden, the Netherlands.
193. Labormedizinisches Zentrum Dr. Risch, Schaan, Liechtenstein.
194. Private University of the Principality of Liechtenstin, Triesen, Liechtenstein.
195. University Insitute of Clinical Chemistry, Inselspital, Bern University Hospital, University of Bern, Bern, Switzerland.
196. Department of Cardiology, University of Groningen, University Medical Center Groningen, Groningen, The Netherlands.
197. Center for Genomic Medicine, Massachusetts General Hospital, Boston, MA, USA.
198. Cardiovascular Research Center, Massachusetts General Hospital, Boston, MA, USA.
201. Cardiovascular Health Research Unit, Departments of Medicine, Epidemiology and Health Services, University of Washington, Seattle, WA, USA.
202. Kaiser Permanente Washington Health Research Institute, Seattle, WA, USA.
203. National Institute for Health Research Imperial Biomedical Research Centre, Imperial College Healthcare NHS Trust and Imperial College London, London, UK.
204. UK Dementia Research Institute (UK DRI) at Imperial College London, London, UK
205. Health Data Research-UK London substantive site, London, U.K

MVP (Million Veteran Program)

MVP Executive Committee

- Co-Chair: J. Michael Gaziano, M.D., M.P.H.

- Co-Chair: Rachel Ramoni, D.M.D., Sc.D.

- Jim Breeling, M.D. (ex-officio)

- Kyong-Mi Chang, M.D.

- Grant Huang, Ph.D.

- Sumitra Muralidhar, Ph.D.

- Christopher J. O’Donnell, M.D., M.P.H.

- Philip S. Tsao, Ph.D.

MVP Program Office

- Sumitra Muralidhar, Ph.D.

- Jennifer Moser, Ph.D.

MVP Recruitment/Enrollment

- Recruitment/Enrollment Director/Deputy Director, Boston – Stacey B. Whitbourne, Ph.D.; Jessica V. Brewer, M.P.H.

- MVP Coordinating Centers

  - Clinical Epidemiology Research Center (CERC), West Haven – John Concato, M.D., M.P.H.
  - Cooperative Studies Program Clinical Research Pharmacy Coordinating Center, Albuquerque - Stuart Warren, J.D., Pharm D.; Dean P. Argyres, M.S.
  - Genomics Coordinating Center, Palo Alto – Philip S. Tsao, Ph.D.
  - Massachusetts Veterans Epidemiology Research Information Center (MAVERIC), Boston - J. Michael Gaziano, M.D., M.P.H.
  - MVP Information Center, Canandaigua – Brady Stephens, M.S.

- Core Biorepository, Boston – Mary T. Brophy M.D., M.P.H.; Donald E. Humphries, Ph.D.

- MVP Informatics, Boston – Nhan Do, M.D.; Shahpoor Shayan

- Data Operations/Analytics, Boston – Xuan-Mai T. Nguyen, Ph.D.

MVP Science

- Genomics - Christopher J. O’Donnell, M.D., M.P.H.; Saiju Pyarajan Ph.D.; Philip S. Tsao, Ph.D.

- Phenomics - Kelly Cho, M.P.H, Ph.D.

- Data and Computational Sciences – Saiju Pyarajan, Ph.D.

- Statistical Genetics – Elizabeth Hauser, Ph.D.; Yan Sun, Ph.D.; Hongyu Zhao, Ph.D.

MVP Local Site Investigators

- Atlanta VA Medical Center (Peter Wilson)

- Bay Pines VA Healthcare System (Rachel McArdle)

- Birmingham VA Medical Center (Louis Dellitalia)

- Cincinnati VA Medical Center (John Harley)

- Clement J. Zablocki VA Medical Center (Jeffrey Whittle)

- Durham VA Medical Center (Jean Beckham)

- Edith Nourse Rogers Memorial Veterans Hospital (John Wells)

- Edward Hines, Jr. VA Medical Center (Salvador Gutierrez)

- Fayetteville VA Medical Center (Gretchen Gibson)

- VA Health Care Upstate New York (Laurence Kaminsky)

- New Mexico VA Health Care System (Gerardo Villareal)

- VA Boston Healthcare System (Scott Kinlay)

- VA Western New York Healthcare System (Junzhe Xu)

- Ralph H. Johnson VA Medical Center (Mark Hamner)

- Wm. Jennings Bryan Dorn VA Medical Center (Kathlyn Sue Haddock)

- VA North Texas Health Care System (Sujata Bhushan)

- Hampton VA Medical Center (Pran Iruvanti)

- Hunter Holmes McGuire VA Medical Center (Michael Godschalk)

- Iowa City VA Health Care System (Zuhair Ballas)

- Jack C. Montgomery VA Medical Center (Malcolm Buford)

- James A. Haley Veterans' Hospital (Stephen Mastorides)

- Louisville VA Medical Center (Jon Klein)

- Manchester VA Medical Center (Nora Ratcliffe)

- Miami VA Health Care System (Hermes Florez)

- Michael E. DeBakey VA Medical Center (Alan Swann)

- Minneapolis VA Health Care System (Maureen Murdoch)

- N. FL/S. GA Veterans Health System (Peruvemba Sriram)

- Northport VA Medical Center (Shing Shing Yeh)

- Overton Brooks VA Medical Center (Ronald Washburn)

- Philadelphia VA Medical Center (Darshana Jhala)

- Phoenix VA Health Care System (Samuel Aguayo)

- Portland VA Medical Center (David Cohen)
- Providence VA Medical Center (Satish Sharma)
- Richard Roudebush VA Medical Center (John Callaghan)
- Salem VA Medical Center (Kris Ann Oursler)
- San Francisco VA Health Care System (Mary Whooley)
- South Texas Veterans Health Care System (Sunil Ahuja)
- Southeast Louisiana Veterans Health Care System (Amparo Gutierrez)
- Southern Arizona VA Health Care System (Ronald Schifman)
- Sioux Falls VA Health Care System (Jennifer Greco)
- St. Louis VA Health Care System (Michael Rauchman)
- Syracuse VA Medical Center (Richard Servatius)
- VA Eastern Kansas Health Care System (Mary Oehlert)
- VA Greater Los Angeles Health Care System (Agnes Wallbom)
- VA Loma Linda Healthcare System (Ronald Fernando)
- VA Long Beach Healthcare System (Timothy Morgan)
- VA Maine Healthcare System (Todd Stapley)
- VA New York Harbor Healthcare System (Scott Sherman)
- VA Pacific Islands Health Care System (Gwenevere Anderson)
- VA Palo Alto Health Care System (Philip Tsao)
- VA Pittsburgh Health Care System (Elif Sonel)
- VA Puget Sound Health Care System (Edward Boyko)
- VA Salt Lake City Health Care System (Laurence Meyer)
- VA San Diego Healthcare System (Samir Gupta)
- VA Southern Nevada Healthcare System (Joseph Fayad)
- VA Tennessee Valley Healthcare System (Adriana Hung)
- Washington DC VA Medical Center (Jack Lichy)
- W.G. (Bill) Hefner VA Medical Center (Robin Hurley)

- White River Junction VA Medical Center (Brooks Robey)

- William S. Middleton Memorial Veterans Hospital (Robert Striker)

eQTLGen

Mawussé Agbessi[1], Habibul Ahsan[2], Isabel Alves[1], Anand Kumar Andiappan[3], Wibowo Arindrarto[4], Philip Awadalla[1], Alexis Battle[5,6], Frank Beutner[7], Marc Jan Bonder[8,9], Dorret I. Boomsma[10], Mark W. Christiansen[11], Annique Claringbould[8,12], Patrick Deelen[8,13,12,14], Tõnu Esko[15], Marie-Julie Favé[1], Lude Franke[8,12], Timothy Frayling[16], Sina A. Gharib[11,17], Greg Gibson[18], Bastiaan T. Heijmans[4], Gibran Hemani[19], Rick Jansen[20], Mika Kähönen[21], Anette Kalnapenkis[15], Silva Kasela[15], Johannes Kettunen[22], Yungil Kim[23,5], Holger Kirsten[24], Peter Kovacs[25], Knut Krohn[26], Jaanika Kronberg[15], Viktorija Kukushkina[15], Zoltan Kutalik[27], Bernett Lee[3], Terho Lehtimäki[28], Markus Loeffler[24], Urko M. Marigorta[18,29,30], Hailang Mei[31], Lili Milani[15], Grant W. Montgomery[32], Martina Müller-Nurasyid[33,34,35], Matthias Nauck[36,37], Michel G. Nivard[38], Brenda Penninx[20], Markus Perola[39], Natalia Pervjakova[15], Brandon L. Pierce[2], Joseph Powell[40], Holger Prokisch[41,42], Bruce M. Psaty[11,43], Olli T. Raitakari[44], Samuli Ripatti[45], Olaf Rotzschke[3], Sina Rüeger[27], Ashis Saha[5], Markus Scholz[24], Katharina Schramm[46,34], Ilkka Seppälä[28], Eline P. Slagboom[4], Coen D.A. Stehouwer[47], Michael Stumvoll[48], Patrick Sullivan[49], Peter A.C. 't Hoen[50], Alexander Teumer[51], Joachim Thiery[52], Lin Tong[2], Anke Tönjes[48], Jenny van Dongen[10], Maarten van Iterson[4], Joyce van Meurs[53], Jan H. Veldink[54], Joost Verlouw[53], Peter M. Visscher[32], Uwe Völker[55], Urmo Võsa[8,15], Harm-Jan Westra[8,12], Cisca Wijmenga[8], Hanieh Yaghootkar[16,56,57], Jian Yang[32,58], Biao Zeng[18], Futao Zhang[32]

Author list is ordered alphabetically

1. Computational Biology, Ontario Institute for Cancer Research, Toronto, Canada
2. Department of Public Health Sciences, University of Chicago, Chicago, United States of America
3. Singapore Immunology Network, Agency for Science, Technology and Research, Singapore, Singapore
4. Leiden University Medical Center, Leiden, The Netherlands
5. Department of Computer Science, Johns Hopkins University, Baltimore, United States of America
6. Departments of Biomedical Engineering, Johns Hopkins University, Baltimore, United States of America
7. Heart Center Leipzig, Universität Leipzig, Leipzig, Germany
8. Department of Genetics, University Medical Centre Groningen, Groningen, The Netherlands
9. European Molecular Biology Laboratory, Genome Biology Unit, 69117 Heidelberg, Germany
10. Netherlands Twin Register, Department of Biological Psychology, Vrije Universiteit Amsterdam, Amsterdam Public Health research institute and Amsterdam Neuroscience, the Netherlands
11. Cardiovascular Health Research Unit, University of Washington, Seattle, United States of America
12. Oncode Institute
13. Genomics Coordination Center, University Medical Centre Groningen, Groningen, The Netherlands
14. Department of Genetics, University Medical Centre Utrecht, P.O. Box 85500, 3508 GA, Utrecht, The Netherlands
15. Estonian Genome Center, Institute of Genomics, University of Tartu, Tartu 51010, Estonia
16. Genetics of Complex Traits, University of Exeter Medical School, Royal Devon & Exeter Hospital, Exeter, United Kingdom
17. Department of Medicine, University of Washington, Seattle, United States of America
18. School of Biological Sciences, Georgia Tech, Atlanta, United States of America
19. MRC Integrative Epidemiology Unit, University of Bristol, Bristol, United Kingdom
20. Amsterdam UMC, Vrije Universiteit, Department of Psychiatry, Amsterdam Public Health research institute and Amsterdam Neuroscience, The Netherlands
21. Department of Clinical Physiology, Tampere University Hospital and Faculty of Medicine and Health Technology, Tampere University, Tampere, Finland
22. University of Helsinki, Helsinki, Finland
23. Genetics and Genomic Science Department, Icahn School of Medicine at Mount Sinai, New York, United States of America

24. Institut für Medizinische InformatiK, Statistik und Epidemiologie, LIFE – Leipzig Research Center for Civilization Diseases, Universität Leipzig, Leipzig, Germany
25. IFB Adiposity Diseases, Universität Leipzig, Leipzig, Germany
26. Interdisciplinary Center for Clinical Research, Faculty of Medicine, Universität Leipzig, Leipzig, Germany
27. Lausanne University Hospital, Lausanne, Switzerland
28. Department of Clinical Chemistry, Fimlab Laboratories and Finnish Cardiovascular Research Center-Tampere, Faculty of Medicine and Health Technology, Tampere University, Tampere, Finland
29. Integrative Genomics Lab, CIC bioGUNE, Bizkaia Science and Technology Park, Derio, Bizkaia, Basque Country, Spain
30. IKERBASQUE, Basque Foundation for Science, Bilbao, Spain
31. Department of Medical Statistics and Bioinformatics, Leiden University Medical Center, Leiden, The Netherlands
32. Institute for Molecular Bioscience, University of Queensland, Brisbane, Australia
33. Institute of Genetic Epidemiology, Helmholtz Zentrum München - German Research Center for Environmental Health, Neuherberg, Germany
34. Department of Medicine I, University Hospital Munich, Ludwig Maximilian's University, München, Germany
35. DZHK (German Centre for Cardiovascular Research), partner site Munich Heart Alliance, Munich, Germany
36. Institute of Clinical Chemistry and Laboratory Medicine, Greifswald University Hospital, Greifswald, Germany
37. German Center for Cardiovascular Research (partner site Greifswald), Greifswald, Germany
38. Department of Biological Psychology, Faculty of Behaviour and Movement Sciences, VU, Amsterdam, The Netherlands
39. National Institute for Health and Welfare, University of Helsinki, Helsinki, Finland
40. Garvan Institute of Medical Research, Garvan-Weizmann Centre for Cellular Genomics, Sydney, Australia
41. Institute of Human Genetics, Helmholtz Zentrum München, Neuherberg, Germany
42. Institute of Human Genetics, Technical University Munich, Munich, Germany
43. Kaiser Permanente Washington Health Research Institute, Seattle, WA, United States of America
44. Centre for Population Health Research, Department of Clinical Physiology and Nuclear Medicine, Turku University Hospital and University of Turku, Turku, Finland
45. Statistical and Translational Genetics, University of Helsinki, Helsinki, Finland
46. Institute of Genetic Epidemiology, Helmholtz Zentrum München - German Research Center for Environmental Health, Neuherberg, Germany
47. Department of Internal Medicine and School for Cardiovascular Diseases (CARIM), Maastricht University Medical Center, Maastricht, The Netherlands
48. Department of Medicine, Universität Leipzig, Leipzig, Germany
49. Department of Medical Epidemiology and Biostatistics, Karolinska Institutet, Stockholm, Sweden
50. Center for Molecular and Biomolecular Informatics, Radboud Institute for Molecular Life Sciences, Radboud University Medical Center Nijmegen, Nijmegen, The Netherlands
51. Institute for Community Medicine, University Medicine Greifswald, Greifswald, Germany
52. Institute for Laboratory Medicine, LIFE – Leipzig Research Center for Civilization Diseases, Universität Leipzig, Leipzig, Germany
53. Department of Internal Medicine, Erasmus Medical Centre, Rotterdam, The Netherlands
54. UMC Utrecht Brain Center, University Medical Center Utrecht, Department of Neurology, Utrecht University, Utrecht, The Netherlands
55. Interfaculty Institute for Genetics and Functional Genomics, University Medicine Greifswald, Greifswald, Germany
56. School of Life Sciences, College of Liberal Arts and Science, University of Westminster, 115 New Cavendish Street, London, United Kingdom
57. Division of Medical Sciences, Department of Health Sciences, Luleå University of Technology, Luleå, Sweden
58. Institute for Advanced Research, Wenzhou Medical University, Wenzhou, Zhejiang 325027, China

BIOS (Biobank-based Integrative Omics Study)

Management Team Bastiaan T. Heijmans (chair)[1], Peter A.C. 't Hoen[2], Joyce van Meurs[3], Aaron Isaacs[4], Rick Jansen[5], Lude Franke[6].

Cohort collection Dorret I. Boomsma[7], René Pool[7], Jenny van Dongen[7], Jouke J. Hottenga[7] (Netherlands Twin Register); Marleen MJ van Greevenbroek[8], Coen D.A. Stehouwer[8], Carla J.H. van der Kallen[8], Casper G. Schalkwijk[8] (Cohort study on Diabetes and Atherosclerosis Maastricht); Cisca Wijmenga[6], Lude Franke[6], Sasha Zhernakova[6], Ettje F. Tigchelaar[6] (LifeLines

Deep); P. Eline Slagboom[1], Marian Beekman[1], Joris Deelen[1], Diana van Heemst[9] (Leiden Longevity Study); Jan H. Veldink[10], Leonard H. van den Berg[10] (Prospective ALS Study Netherlands); Cornelia M. van Duijn[4], Bert A. Hofman[11], Aaron Isaacs[4], André G. Uitterlinden[3] (Rotterdam Study).

Data Generation Joyce van Meurs (Chair)[3], P. Mila Jhamai[3], Michael Verbiest[3], H. Eka D. Suchiman[1], Marijn Verkerk[3], Ruud van der Breggen[1], Jeroen van Rooij[3], Nico Lakenberg[1].

Data management and computational infrastructure Hailiang Mei (Chair)[12], Maarten van Iterson[1], Michiel van Galen[2], Jan Bot[13], Dasha V. Zhernakova[6], Rick Jansen[5], Peter van 't Hof[12], Patrick Deelen[6], Irene Nooren[13], Peter A.C. 't Hoen[2], Bastiaan T. Heijmans[1], Matthijs Moed[1].

Data Analysis Group Lude Franke (Co-Chair)[6], Martijn Vermaat[2], Dasha V. Zhernakova[6], René Luijk[1], Marc Jan Bonder[6], Maarten van Iterson[1], Patrick Deelen[6], Freerk van Dijk[14], Michiel van Galen[2], Wibowo Arindrarto[12], Szymon M. Kielbasa[15], Morris A. Swertz[14], Erik. W van Zwet[15], Rick Jansen[5], Peter-Bram 't Hoen (Co-Chair)[2], Bastiaan T. Heijmans (Co-Chair)[1].

1. Molecular Epidemiology Section, Department of Medical Statistics and Bioinformatics, Leiden University Medical Center, Leiden, The Netherlands
2. Department of Human Genetics, Leiden University Medical Center, Leiden, The Netherlands
3. Department of Internal Medicine, ErasmusMC, Rotterdam, The Netherlands
4. Department of Genetic Epidemiology, ErasmusMC, Rotterdam, The Netherlands
5. Department of Psychiatry, VU University Medical Center, Neuroscience Campus Amsterdam, Amsterdam, The Netherlands
6. Department of Genetics, University of Groningen, University Medical Centre Groningen, Groningen, The Netherlands
7. Department of Biological Psychology, VU University Amsterdam, Neuroscience Campus Amsterdam, Amsterdam, The Netherlands
8. Department of Internal Medicine and School for Cardiovascular Diseases (CARIM), Maastricht University Medical Center, Maastricht, The Netherlands
9. Department of Gerontology and Geriatrics, Leiden University Medical Center, Leiden, The Netherlands
10. Department of Neurology, Brain Center Rudolf Magnus, University Medical Center Utrecht, Utrecht, The Netherlands
11. Department of Epidemiology, ErasmusMC, Rotterdam, The Netherlands
12. Sequence Analysis Support Core, Leiden University Medical Center, Leiden, The Netherlands
13. SURFsara, Amsterdam, the Netherlands
14. Genomics Coordination Center, University Medical Center Groningen, University of Groningen, Groningen, the Netherlands
15. Medical Statistics Section, Department of Medical Statistics and Bioinformatics, Leiden University Medical Center, Leiden, The Netherlands

References

1. Purcell S, Neale, B.; Todd-Brown, K.; Thomas, L.; Ferreira, M.A.R.; Bender, D.; Maller, J.; Sklar, P.; de Bakker, P.I.W.; Daly, M.J.; et al. PLINK: A Tool Set for Whole-Genome Association and Population-Based Linkage Analyses. Am. J. Hum. Genet. 2007, 81, 559 – 575.
2. 1000 Genomes Project Consortium; Auton, A.; Brooks, L.D.; Durbin, R.M.; Garrison, E.P.; Kang, H.M.; Korbel, J.O.; Marchini, J.L.; McCarthy, S.; McVean, G.A.; et al. A Global Reference for Human Genetic Variation. Nature 2015, 526, 68–74, doi:10.1038/nature15393.
3. Wang, K.; Li, M.; Hakonarson, H. ANNOVAR: Functional Annotation of Genetic Variants from High-Throughput Sequencing Data. Nucleic Acids Res. 2010, 38, e164–e164, doi:10.1093/nar/gkq603.
4. McLaren, W.; Gil, L.; Hunt, S.E.; Riat, H.S.; Ritchie, G.R.S.; Thormann, A.; Flicek, P.; Cunningham, F. The Ensembl Variant Effect Predictor. Genome Biol. 2016, 17, doi:10.1186/s13059-016-0974-4.
5. Battle, A.; Mostafavi, S.; Zhu, X.; Potash, J.B.; Weissman, M.M.; McCormick, C.; Haudenschild, C.D.; Beckman, K.B.; Shi, J.; Mei, R.; et al. Characterizing the Genetic Basis of Transcriptome Diversity through RNA-Sequencing of 922 Individuals. Genome Res. 2014, 24, 14–24, doi:10.1101/gr.155192.113.
6. Barbeira, A.N.; Dickinson, S.P.; Bonazzola, R.; Zheng, J.; Wheeler, H.E.; Torres, J.M.; Torstenson, E.S.; Shah, K.P.; Garcia, T.; Edwards, T.L.; et al. Exploring the Phenotypic Consequences of Tissue Specific Gene Expression Variation Inferred from GWAS Summary Statistics. Nat. Commun. 2018, 9, 1825, doi:10.1038/s41467-018-03621-1.
7. Giambartolomei, C.; Vukcevic, D.; Schadt, E.E.; Franke, L.; Hingorani, A.D.; Wallace, C.; Plagnol, V. Bayesian Test for Colocalisation between Pairs of Genetic Association Studies Using Summary Statistics. PLoS Genet. 2014, 10, e1004383, doi:10.1371/journal.pgen.1004383.
8. Võsa, U.; Claringbould, A.; Westra, H.-J.; Bonder, M.J.; Deelen, P.; Zeng, B.; Kirsten, H.; Saha, A.; Kreuzhuber, R.; Yazar, S.; et al. Large-Scale Cis- and Trans-EQTL Analyses Identify Thousands of Genetic Loci and Polygenic Scores That Regulate Blood Gene Expression. Nat. Genet. 2021, 53, 1300–1310, doi:10.1038/s41588-021-00913-z.
9. Zhu, Z.; Zhang, F.; Hu, H.; Bakshi, A.; Robinson, M.R.; Powell, J.E.; Montgomery, G.W.; Goddard, M.E.; Wray, N.R.; Visscher, P.M.; et al. Integration of Summary Data from GWAS and EQTL Studies Predicts Complex Trait Gene Targets. Nat. Genet. 2016, 48, 481–487, doi:10.1038/ng.3538.
10. McRae, A.F.; Marioni, R.E.; Shah, S.; Yang, J.; Powell, J.E.; Harris, S.E.; Gibson, J.; Henders, A.K.; Bowdler, L.; Painter, J.N.; et al. Identification of 55,000 Replicated DNA Methylation QTL. Sci. Rep. 2018, 8, doi:10.1038/s41598-018-35871-w.

11. Battram, T.; Yousefi, P.; Crawford, G.; Prince, C.; Babei, M.S.; Sharp, G.; Hatcher, C.; Vega- Salas, M.J.; Khodabakhsh, S.; Whitehurst, O.; et al. The EWAS Catalog: A Database of Epigenome-Wide Association Studies 2021.
12. Eales, J.M.; Jiang, X.; Xu, X.; Saluja, S.; Akbarov, A.; Cano-Gamez, E.; McNulty, M.T.; Finan, C.; Guo, H.; Wystrychowski, W.; et al. Uncovering Genetic Mechanisms of Hypertension 27 | P a g e through Multi-Omic Analysis of the Kidney. Nat. Genet. 2021, 53, 630–637, doi:10.1038/s41588-021-00835-w.
13. Roadmap Epigenomics Consortium; Kundaje, A.; Meuleman, W.; Ernst, J.; Bilenky, M.; Yen, A.; Heravi-Moussavi, A.; Kheradpour, P.; Zhang, Z.; Wang, J.; et al. Integrative Analysis of 111 Reference Human Epigenomes. Nature 2015, 518, 317–330, doi:10.1038/nature14248.
14. The ENCODE Project Consortium An Integrated Encyclopedia of DNA Elements in the Human Genome. Nature 2012, 489, 57–74, doi:10.1038/nature11247. 15. Zerbino, D.R.; Wilder, S.P.; Johnson, N.; Juettemann, T.; Flicek, P.R. The Ensembl Regulatory Build. Genome Biol. 2015, 16, doi:10.1186/s13059-015-0621-5.
15. Zerbino, D.R.; Wilder, S.P.; Johnson, N.; Juettemann, T.; Flicek, P.R. The Ensembl Regulatory Build. Genome Biol. 2015, 16, doi:10.1186/s13059-015-0621-5.
